# Agriculture, Aid, and Economic Growth in Africa

**DOI:** 10.1093/wber/lhx029

**Published:** 2018-06-13

**Authors:** John W. McArthur, Jeffrey D. Sachs

**Affiliations:** 1 Senior Fellow at the Brookings Institution; 2 University Professor at Columbia University

**Keywords:** O11, O21, O41, O55, Q10, Africa, agriculture, growth, foreign aid

## Abstract

How can foreign aid to agriculture support economic growth in Africa? This paper constructs a geographically indexed applied general equilibrium model that considers pathways through which aid might affect growth and structural transformation of labor markets in the context of soil nutrient variation, minimum subsistence consumption requirements, domestic transport costs, labor mobility, and constraints to self-financing of agricultural inputs.Using plausible parameters, the model is presented for Uganda as an illustrative case.We present three stylized scenarios to demonstrate the potential economy-wide impacts of both soil nutrient loss and replenishment, and how foreign aid can be targeted to support agricultural inputs that boost rural productivity and shift labor to boost real wages. One simulation shows how a temporary program of targeted official development assistance (ODA) for agriculture could generate, contrary to traditional Dutch disease concerns, an expansion in the primary tradable sector and positive permanent productivity and welfare effects, leading to a steady decline in the need for complementary ODA for budget support.

## 1 Introduction

How can foreign aid support economic growth and poverty reduction in sub-Saharan Africa (hereafter “Africa”)? An assessment of this important question can begin by recognizing three points. First, the majority of Africa’s extremely poor people still live in rural areas and primarily work on smallholder subsistence farms for their livelihoods. These settings are categorized by low and slow-growing agricultural value added per worker, low staple crop yields, soil nutrient depletion, and low levels of modern input use (Stoorvogel and Smaling 1990; McArthur 2015). Yet input technologies now exist—such as fertilizer, modern seeds, land management, and small-scale irrigation—to boost productivity in these areas. Among other factors, Malawi’s post-2005 results in doubling average national maize yields through an aid-supported input subsidy program prompted considerable analysis regarding the potential merits of increasing public finance to support small-holder agriculture throughout Africa (e.g., Diao, Headey, and Johnson 2008 Duflo, Kremer, and Robinson 2011; Chirwa and Dorward 2013; Jayne and Rashid 2013).

Second, there is considerable evidence indicating that agricultural growth has had important aggregate effects in reducing global poverty, especially extreme poverty (Bourguignon and Morrison 1998; Gollin, Parente, and Rogerson 2002; Christiaensen, Demery, and Kuhl 2011). Some researchers posit that the role of agriculture has been fundamental, if underappreciated, in promoting growth in non-agricultural sectors, including through channels of structural transformation from low-productivity rural sectors to higher- productivity urban sectors (e.g., Bezemer and Heady 2008; de Janvry and Sadoulet 2009; McArthur and McCord 2017). However, the precise channels through which public investments in agriculture might promote sectoral outcomes remain inadequately understood, prompting some researchers to caution against prioritizing agriculture compared to other sectors (Collier and Dercon 2013; Dercon and Gollin 2014).

Third, an extensive cross-country empirical literature has long grappled to specify the conditions and pathways through which aid, as a source of public finance, might support growth (e.g., Hansen and Tarp 2001; Werker, Ahmed, and Cohen 2009; Arndt, Jones, and Tarp 2015; Galiani et al. 2017). A subset of that literature has focused on such questions in the African context (e.g., Collier and Gunning 1999; Sachs et al. 2004; Gomanee, Girma, and Morrissey 2005). Econometrically, one of the core challenges is to distinguish between the respective purposes of different types of aid. Clemens et al. (2012), for example, separate out “early impact” aid that supports sectors like roads, energy, agriculture, and industry, any of which might be expected to boost growth in the short to medium term. This is distinguished from other social sector activities like education, health, water, and humanitarian assistance, “whose growth effect might arrive far in the future or not at all” (p. 599). The authors find a positive average relationship between aid and growth, but the results have been questioned by Roodman (2014), leaving room for argument.

These debates remain important, but their common emphasis on cross-country empirical relationships can only provide limited insight regarding the actual economic channels through which aid might support growth, poverty reduction, and labor’s structural transformation toward higher-productivity sectors in countries where the majority of people still live in rural areas, and where the primary economic activity remains staple crop farming. Even among “early impact” channels, aid for agriculture might initiate different structural dynamics than aid aiming to support energy systems or manufacturing. Moreover, given the emphasis that agronomic science has also placed on the central importance of soil nutrients to increasing African agricultural productivity (Stoorvogel, Smaling, and Janssen 1993; Sanchez 2010), it is highly relevant to consider how soil nutrient dynamics interact with broader agricultural and economic dynamics. There is therefore merit in considering the channels through which publicly financed agricultural input support programs, potentially backed by foreign aid, could generate economy-wide outcomes. That is the aim of this paper.

To explore these dynamics, the paper introduces a simulation model for considering how soil nutrient loss can promote stagnation in a predominantly rural African subsistence economy and, conversely, how green revolution-type input support (i.e., aiming at a major increase in smallholder productivity) can prompt accelerated labor shifts across tradable and nontradable sectors. In the model, farms also need to meet a minimum subsistence level of food production, which is linked to potentially variable soil nutrient balances across geographies. A public subsidy helps overcome farm-level constraints to self-financing of inputs. Most low-income country governments cannot afford to finance an input package through their own budget envelopes, so the model assumes this to be financed by official development assistance (ODA). A distinction is drawn between this ODA targeted for agricultural inputs and other “cash” ODA allocated to general budget support.

A green revolution-type agricultural productivity boost in the form of a doubling or more of staple crop yields would mark a tremendous direct supply-side structural change in a typical African economy. Because cereals and other staple foods in subsistence economies are mainly consumed on farms and in local markets, they are overwhelmingly nontradable goods with locally determined prices. A boost in supply should have strong deflationary pressures for the majority of the population’s main consumption good while also spurring the allocation of labor and investment in an export-oriented cash crop sector. Therefore, unlike ODA for consumption or for investments with small supply-side effects, ODA increases to support an African green revolution might have anti-Dutch disease effects, contrary to the concerns of Rajan and Subramanian (2008, 2011).

The paper builds on the logic presented in Adam and Bevan’s (2006) careful consideration of aid’s supply-side productivity effects in a model calibrated to Uganda. In a migration-free model with Engel curve attributes, they focus on public-infrastructure-induced productivity spillovers and learning by doing in the export sector. Their model shows that welfare effects and real exchange rate dynamics are highly sensitive to the location of productivity effects and the composition of domestic demand. It emphasizes aggregate linkages to rural productivity in agricultural sectors, but does not explore these dynamics in detail.

We take up that challenge by building a subsistence threshold-based framework that shows how a poverty-trap dynamic can take shape in the presence of low-input agriculture and soil nutrient depletion. The model presented here does not aim to provide specific empirical results or point estimates. Instead, in line with the arguments of Robinson and Lofgren (2005), it aims to outline directions of medium-term structural shifts. Some aspects are similar to the nontradable agriculture analytical model in Matsuyama (1992), although here staple foods are treated as nontradable due to the reality of subsistence food economies with low private and public capital stocks, rather than as a function of overall economy openness. Indeed, one important part of our model is the ability for labor to shift easily between nontradable (food) and tradable (cash crop) sectors while remaining on farm.

The approach presented here differs from Lofgren, Harris, and Robinson (2002), who follow the Dervis, de Melo, and Robinson (1982) tradition of a standardized, mixed-complementarity computable general equilibrium (CGE) model, including a monolithic and exogenous public sector.^1^ It also differs from the Poverty and Economic Policy Research Network (PEP) standard model (Decaluwe et al. 2009), which has a nested production structure and excludes home consumption, and from other Africa-focused macroeconomic models that have emphasized social development outcomes. For example, Agenor and colleagues (Agenor, Bayraktarb, and El Aynaoui 2005; Agenor, Bayraktarb, and Pinto 2005) and Pinto and Bayraktarb (2005) model the real economy through a single representative sector with a parameterized elasticity on poverty.

The Maquette for Millennium Development Goal Simulations (MAMS) model originally developed by Bourguignon et al. (2004) was novel for its decomposition of government sectors, emphasizing interactions between labor markets, infrastructure, and the achievement of outcome targets for poverty, education, health and water, and sanitation. Its major contribution is the ability to show the evolution of intermediate outcomes en route to internationally agreed development goals and highlight the implications of sequencing investments among sectors (Bourguignon and Sundberg 2006a, 2006b). The original MAMS model had a single representative productive sector, which did not permit evaluation of subsistence dynamics, although more recent applications have incorporated the core Lofgren, Harris, and Robinson (2002) framework as the basis for evaluating more detailed analysis of productive sectors (Lofgren, Cicowiez, and Diaz-Bonilla 2013).

Some previous models have integrated the biophysical aspects of agricultural productivity into a developing-country CGE framework. For example, Alfsen et al. (1997) use Aune and Lal’s (1995) Tropical Soil Productivity Calculator in a 17-sector closed public sector model to show the contribution of soil nutrients to the growth of gross domestic product (GDP). A limitation of that model is that it does not include a ceiling for possible soil nutrient accumulation and does not allow for the practical reality of zero fertilizer use among large numbers of small-holder farmers, because the fertilizer term enters as a simple input in a Cobb-Douglas production function and zero input implies zero output. Wiig et al. (2001) pursued a comparable strategy to introduce soil degradation as a time-dependent Hicks-neutral productivity coefficient in the agricultural production functions. Meanwhile Holden, Shiferaw, and Pender (2005) add soil nutrient dynamics to a sophisticated multi-product household production and welfare assessment in the Ethiopian highlands.

The most similarly green revolution-spirited CGE approach to the model in this paper is presented by Breisinger et al. (2011), who extend the approach of Lofgren, Harris, and Robinson (2002) to include within-country disaggregation by agroecological zone, crop market, and income group. Their model is applied to Ghana, and a green revolution is achieved through exogenously defined total factor productivity improvements to achieve target yields, prompting greater input use through factor markets. Foreign savings are fixed, so incremental investments are all financed through domestic resources.

Our model illustrates pathways through which targeted investments in agriculture, supported by ODA, could plausibly promote structural transformation in Africa. Indicative parameters for Uganda are used to show representative dynamics. The economy is suitable for analysis because its labor is still overwhelmingly in rural areas and remains largely focused on staple food production. Rural productivity remains extremely low, and a considerable share of the country still lives in extreme poverty. Spread across four major regions, the country’s agricultural systems have major variations across local climatic zones, soil types, and soil nutrient flows over time. Soil nutrient losses have been considerable, and nutrient stocks have fallen below critical levels in many parts of the country.

The rest of the paper proceeds in four sections. Following this introduction, section 2 presents the general equilibrium model. Section 3 presents a range of scenarios using the model. Section 4 discusses some key insights generated by the simulations. A final section concludes.

## 2 Model Description

The model has several key attributes aligned with many low-income African economies. The first is a dominant factor of rural subsistence economic stagnation, with low savings and flat incomes in the absence of productivity increases in agriculture. The second is a minimum subsistence food requirement that underpins the thresholds for agricultural diversification, savings, and labor switching to other sectors. The third is a soil nutrient depletion and accumulation process that directly feeds into agricultural production functions. The fourth is labor mobility from rural to urban areas, with migration parameterized to respond to relative wages.

The fifth relevant attribute is a constraint to self-financing agricultural inputs, like fertilizer, among small-holder farmers. The sixth is a road-building component and Samuelson-style “iceberg” transport cost structure that directly affects relative prices for agricultural outputs and decreases in the presence of road improvement. The seventh attribute is the possibility of geographic variation in productivity levels and locations of production, including variation in soil nutrient flows and transport infrastructure.

The model follows a recursive structure over 10 periods, with decisions depending on past and present periods but no forward-looking dynamics. The productive economy includes both tradable and nontradable sectors, with no intermediate goods. The nontradable sectors are staple food production, rural services, and urban services, all of which have locally determined prices. The two domestic tradable sectors are cash crops and manufacturing, both of which are assumed to have fixed numeraire tradable prices, zero domestic consumption, and infinite elasticity of demand. In reality, Uganda’s manufacturing sector is very small and includes a focus on import substitution, so the assumption of full export orientation is made for the purposes of simplification within the model’s core focus on rural and rural-urban dynamics. Implicit in the model is a fixed nominal exchange rate, so changes in domestic prices indicate changes in the real exchange rate. There is also an imported sector that provides consumption goods and capital goods for investment with infinitely elastic supply at fixed prices.

The model is constructed to permit flexibility around the number of urban and rural geographic units. Simulations presented here include four rural units—mapping to Uganda’s Eastern, Western, Central, and Northern regions—plus one urban unit, Kampala. Historically Kampala has accounted for approximately 85 percent of the country’s urban economic activity, so this is assumed to be a reasonable simplification of the underlying national economy. The two agricultural sectors and the rural service sector are only active in the model’s rural areas. Manufacturing and the urban service sector only take place in the city. Food is produced in the rural sectors to feed both the local rural population and the urban population. 1 describes the geographic indexing of productive sector activities. 2 shows the corresponding information for public sector activities.

**Table 1:** Geographic Indexing of Productive Sector Activity

Sector	Price(T/NT)	Rural regions				Urban region
		Western	Eastern	Northern	Central	Kampala
Staple food	NT	✓	✓	✓	✓	
Cash crops	T	✓	✓	✓	✓	
Rural service	NT	✓	✓	✓	✓	
Urban service	NT					✓
Manufacturing	T					✓
Imported capital & consumption goods	T	✓	✓	✓	✓	✓

*Source*: Authors.

*Note:* T = tradable; NT = nontradable.

**Table 2:** Geographic Indexing of Public Sector Activities

	Rural regions				Urban region
	Western	Eastern	Northern	Central	Kampala
Agriculture (green revolution package)	✓	✓	✓	✓	
Roads	✓	✓	✓	✓	
Other services	✓	✓	✓	✓	✓
Administration					✓

*Source:* Authors.

The model’s emphasis on Engel’s law and non-homothetic preferences links directly to its rural-urban divide. Rural staple food production must satisfy a minimum level of aggregate per capita food requirements for both rural and urban populations. A savings-driven neoclassical closure applies a fixed savings rate on incomes above the minimum food basket, and private saving is set equal to productive sector investment. Capital is immobile across regions, although foreign investment is possible in the urban manufacturing sector.

In addition to the option of supporting agricultural inputs, the formal public sector includes considerable rural road building alongside urban public administration and other services like health and education. The government’s fiscal balance is financed by external ODA “cash,” equivalent to budget support. This budget support is fully separate from ODA-financed agricultural inputs, which are imported on a pre-planned basis across a 10-year time horizon. The endogeneity of cash ODA differs from similar models that typically frame official foreign savings as fixed. This allows comparison of ODA implications for different scenarios. The model structure allows the option to integrate both capital and recurrent government spending accounts, with the possibility for multi-year budget variations by geographic zone, subsector, and import content.

Labor is fully mobile from rural to urban regions, although not across rural regions, and responds to relative real incomes. Total labor is fixed. Implicit in the model is an assumption that all labor has a home rural region and that each rural region has a fixed total implicit population of origin, so even the labor present in the urban area in the first period is linked to one of the four rural areas. In allocating their labor, rural households not directly hired by the public sector can choose between working in staple foods, cash crops, rural services, or migrating to the urban area, where manufacturing and urban services are the productive sector options. Prices are free variables playing market clearing functions, with rural prices segmented from urban prices.

Real incomes, net of food, are equilibrated instantaneously in the rural sectors and over time between rural and urban sectors. Thus, the most fundamental impulses driving labor markets are productivity in staple foods, relative food prices between rural and urban areas, and real income differences between rural and urban areas. Food prices are affected by transport costs, which are in turn determined by the scale of the road network. As the road network increases, transport losses decrease and less total food production is required to feed the population.

The full details of the model are presented in the supplementary online appendix, including all equations, variables, and parameters. Here we highlight some of the most important structural equations and dynamics. As a notation convention, model variables are listed in *>CAPITAL*> letters and parameters are listed in lowercase italics (e.g., *theta*).

### Agricultural Production

The model captures basic soil nutrient dynamics in a manner that allows soil-relevant estimation of yields with relative simplicity while focusing on key policy decision variables. As shown in EQ.1.1 and EQ.1.3 the agricultural production functions for food, *F,* and cash crops, CC, are Cobb-Douglas and indexed to each rural region.^2^ Land is considered a fixed parameter in the immediate term and presented without an exponent, as are the *theta* productivity terms. The coefficients on capital and labor therefore sum to less than 1, and labor (here *LF* and *LCC*) serves as the market clearing factor. Agricultural diversification is achieved when labor shifts from staple crops to cash crops.

There are two elements of note in the agricultural equation structure. The first is the soil productivity parameter, *S,* which itself follows a logistic functional form in EQ.1.5, with the numerator defined as an upper-bound level of soil productivity (*uppersoil*). The denominator is partly determined by the slope of the underlying S-curve, set by *rho,* which has a value of 1.5 in the simulations. (Values greater than 1 imply steepness closer to the top of the curve.) The other part of the denominator is determined by *EXPNUT,* which represents the exponential of cumulative recent nutrient flows *NUTSUM,* as per equation EQ.1.6. The net nutrient flows in a period *(NETIN)* are defined in EQ.1.9 by the inflow of external inputs alongside an exogenous rate of nutrient loss, *nlossrate*.^3^

The second element to note in EQ.1.1 and EQ.1.3 is that agricultural inputs enter the equation as a single (1 + *INPUT)* linear term in order to accommodate the common African scenario of zero initial modern input use, especially fertilizer. Conceptually, the *INPUT* term represents Leontief-style complementarities among a package of yield-boosting inputs, such as fertilizer, fertilizer-responsive seeds, and inputs for land management that might reduce erosion and promote soil-based “green water” efficiency (Rockstrom et al. 2009). The *INPUT* term therefore has a dual effect. It provides both a direct productivity boost in the current year’s output, as indicated directly in the production function, and an indirect boost through the following year’s underlying soil productivity.

Farmers are implicitly choosing to allocate their labor among food and cash crops within each period, while using the same input mix across both types of crops and allocating investments only to cash crop capital (KCC). Water is a crucial factor for agriculture too, but given the rain-fed nature of most African agriculture and the order-of-magnitude larger capital outlays required even for small-scale “blue water” irrigation compared to “green water” (ibid.), we assume those to be incorporated in *KCC.*

(EQ.1.1) $$F_{i,t}=S_{i,t}*thetaf_{i}*land_{i}*\left(1+INPUT_{i,t}\right)*LF_{i,t}^{alpha\;f}*kf_{i}^{beta\;f}$$

(EQ.1.3) $$CC_{i,t}=S_{{}_{i,t}}^{*}thetaacc_{i}*land_{i}*\left(1+INPUT_{i,t}\right)*LCC_{i,t}^{alphacc}*{\left(kcsale*KCC_{i,t}\right)}^{betacc}$$

(EQ.1.5) $$S_{i,t}=\frac{uppersoil_{i}}{1+rho*EXPNUT_{i,t}}$$

(EQ.1.6) $$EXPNUT_{i,t}=e^{-NUTSUM_{i,t}}$$

(EQ.1.7) $$NUTSUM_{i,t+1}=NUTSUM_{i,t}+NETIN_{i,t}$$

(EQ.1.9) $$NETIN_{i,t}=INPUT_{i,t}-nlossrate$$

(EQ.1.10) $$INPUT_{i,t}=min\left(1,max\left(0,\left(kcscale*\left(KCC_{i,t}-\left(\left(1+khurdle\right)*kcc0_{i}\right)\right)\right)\right)+greenoda_{t}\right)$$

The equation for input use (EQ.1.10) is central to the model. The amount of input use takes a value ranging from zero to one, meaning that the (1 + INPUT) multiplier term in EQ.1.1 and EQ.1.3 can take an absolute value ranging from one to two. Market-purchased input use is restricted by a household poverty constraint. We deploy a minimum capital requirement in cash crops, *khurdle,* to reflect a wealth level required to afford inputs at the beginning of a planting season and bear the risk of adverse farm shocks, in addition to a collateral requirement for borrowing. Alternative income-based constraints to purchasing inputs could also be deployed without significantly changing the model’s core dynamics.

The final term in EQ.1.10, *greenoda,* represents the ODA-financed package of green revolution- supportive inputs, as a supplement to the market-based input purchases. The agricultural inputs are all presumed to be imported with fixed numeraire prices, so the *greenoda* variable can be interpreted as representing both a physical amount and a financial amount. As mentioned earlier, this targeted agricultural ODA is different from the budget gap-filling aid for budget support *(CASHODA)* described below, since the agricultural ODA is set in advance as an exogenous policy parameter for each period. This allows for a multi-year input support program that can also have a pre-committed phase-out over time.

### Food Market

Food market-clearance helps drive the model too. Urban food demand in Kampala, *FKAMP,* is the product of urban labor, *LU,* and food requirements per capita, *phi* (EQ.3.1). This *phi* parameter is also pivotal to the model’s dynamics. Conceptually, it represents an amalgam of physical units across staple crops.^4^ For purposes of intuition, this can be considered as the volume representing a required minimum of staple food calories per capita. In the food markets, urban food supply, which equals urban food demand, is defined as the sum of all rural areas’ food surpluses minus the losses *(TLOSS)* incurred in transporting food from the rural areas to the urban areas (EQ.3.2). Each rural region’s food surplus *(FSURP)* is defined as its food production minus the product of the region’s labor size and food requirements per capita (EQ.3.3). The on-farm crop choice optimization occurs by equilibrating the marginal product of cash crops and the marginal product of staple food (EQ.5.8 in supplementary online appendix).

(EQ.3.1) $$FKAMP_{t}=phi*LU_{t}$$

(EQ.3.2) $$FKAMP_{t}=\sum_{i}FSURP_{i,t}*\left(1-TLOSS_{i,t}\right)$$

(EQ.3.3) $$FSURP_{i,t}=F_{i,t}-phi*LR_{i,t}$$

### Transport Costs and Prices

Iceberg transport cost adjustments help drive the model’s allocation of labor and production across sectors and among rural and urban areas. Agricultural transport losses from rural to urban areas are an inverse function of the rural road stock, with initial transport losses indexed to each region (see EQ.4.1 in supplementary online appendix). The urban price of food is more expensive than the rural price of food by an amount scaled to the degree of transport losses (see EQ.4.2 in the supplementary online appendix). The farm-gate price of cash crops, as internationally priced goods, has a similar inverse relation to transport costs. The manufactured good and imported agricultural inputs are assumed to have a sufficiently high value per weight that domestic transport costs are not significant.^5^

### Labor Income and Migration

The real wage for urban services *(RWSU)* and the manufacturing sector *(RWMAN)* are set as the nominal wage minus the cost of urban food requirements, all adjusted for the non-food urban consumer price index, *CPINFU,* as in EQ.5.10. The real rural service wage is set similarly using each rural region’s local food price and non-food price index, *CPINFR* (see EQ.5.11 in supplementary online appendix). As shown in EQ.5.12, the real per capita farm income, *YFARM,* includes the sum of agricultural households’ food crop income *(VF)* plus the value added from cash crop income (VCC), minus the equivalent cost of the minimum food need for farm workers *(LFARM)* and the cost of market-purchased inputs.

(EQ.5.10) $$RWSU_{t}\frac{WSU_{t}-phi*PFU_{t}}{CPINFU_{t}}$$

(EQ.5.12) $$YFARM_{i,t}=\frac{VF_{i,t}+VCC_{i,t}-phi*PFR_{i,t}*LFARM_{i,t}-\left(INPUT_{i,t}-greenoda_{t}\right)}{CPINFR_{i,t}*LFARM_{i,t}}$$

The urban labor equilibrium is set by equating the real wage in manufacturing with the real wage in urban services. Within rural regions, real service wages are equated with real farm incomes, and mobility is instantaneous across sectors. Migration is a function of rural-urban wage differentials, which are minimized over time due to migration.

### Disposable Income, Savings, and Consumption

Disposable income is set as the sum of sector incomes net of staple food consumption, market-based agricultural input purchases, and taxes (see EQ.7.1 and EQ.7.2 in supplementary online appendix). The savings equations assume fixed savings rates within urban and rural areas, respectively, set as a fraction of disposable income (see EQ.7.5 and EQ.7.6 in the supplementary online appendix). The model also assumes a minimum level of non-food consumption, *cmin,* composed of both local services and imported goods. If disposable incomes are below *cmin,* then saving is zero. Consistent with the possibility of a savings-based poverty trap, the net savings rate therefore increases as households cross an average income threshold that pays for both minimum food needs and the minimum consumption basket.

### External Balance

Total exports are equal to total cash crop production (net of domestic transport losses) plus total manufacturing production. Urban private investment imports are equal to the sum of urban saving plus all foreign direct investment, which is in turn determined by the difference between the local rate of return and the exogenous global rate of return. Rural private investment imports are equal only to rural savings. Public investment goods for roads and other social sectors are imported, so total imports *(IMPORT)* are given in EQ.8.6 as the sum of agricultural input use; imported rural and urban consumption goods *(IMPRC* and *IMPUC);* imported rural and urban investment goods *(IMPRI* and *IMPUI);* plus government imports for general investment *(PUBINV),* road investment *(ROADINV),* and commodities *(TOTIMPG).*

(EQ.8.6) $$IMPORT_{i}=\sum_{i}INPUT_{i,t}+\sum_{i}IMPRC_{i,t}+IMPUC_{t}+\sum_{i}IMPRI_{i,t}+IMPUI_{t}+PUBINV_{t}+ROADINV_{t}+TOTIMPG_{t}$$

### Public Sector and Government Balance

Government tax revenues *(TAX)* are collected on after-food incomes across all sectors, including urban and rural government worker incomes (see EQ.10.1 in supplementary online appendix). Total public expenditures *(TOTEXP)* are financed by tax revenues and budget support, *CASHODA,* as shown in EQ10.2. As mentioned, this budget support ODA is distinct from the ODA for targeted agricultural inputs. Total public expenditure is the sum of both recurrent expenditure and capital expenditure, including road- building, which affects transport costs.^6^ Recurrent public sector costs are defined as the sum of labor costs and commodities and are assumed to include operations and maintenance.

(EQ.10.2) $$TOTEXP_{t}=TAX_{t}+CASHODA_{t}$$

## 3 Scenarios

We present three stylized scenarios to illustrate the model’s core dynamics. As shown in 3, the first scenario provides an analytical baseline. It includes no soil productivity parameter and no targeted ODA for agriculture, so movements over 10 years are primarily driven by savings, investment, and depreciation across sectors. The second scenario introduces the soil productivity and nutrient flow parameters in the agricultural production functions in order to examine how nutrient losses might affect broader outcomes. The third scenario builds on the second by introducing targeted ODA for agricultural inputs. We dub this the “green revolution” scenario, recognizing that this is an analytical simplification of the actual mix of activities that might enable an African green revolution in practice. The external support program starts in Year 3, and then is gradually phased out over the following five years, such that it decreases to zero for Years 8, 9, and 10. All scenarios include the same annual improvements in the road network.

The model is applied using General Algebraic Modeling System (GAMS) software and the “CONOPT” nonlinear solver. Indicative parameters are set to match the general nature of the Ugandan economy as of the mid-2000s (see supplementary online appendix S4). More precise and detailed country-specific refinements could be applied as desired. For the purposes of the illustrative scenarios presented here, the model is structured to match the country’s four main regions. Kampala is located within the Central region, so Central has the lower initial transport loss when sending food to the urban area.

**Table 3:** Mapping of Key Model Components, by Scenario

Key model component	Scenario		
	1	2	3
	Baseline	Soil productivity	Green revolution
Soil productivity term in agricultural production functions		✓	✓
Green revolution input package introduced in years 3-7			✓

*Source:* Authors.

For each scenario, 4 reports values for a selection of key variables, including: soil productivity, modern agricultural input use, labor movements, prices, real wages, savings, capital stocks, government aggregates, external balances, aggregate aid flows, and total private output (i.e., excluding the government wage bill) indexed to first-period prices. Specifying unit measures can commonly present a challenge in an analytically distilled CGE model like this one. For purposes of intuition, each labor unit can be roughly interpreted as representing a million workers, while prices and economic aggregates can be interpreted, as previously mentioned, in domestic currency terms relative to a fixed nominal exchange rate and constant numeraire tradable prices. Among the rural zones, 4 only presents Western region values for the sake of space. Each region follows slightly different dynamics, guided by initial conditions, but any region’s values demonstrate similar trends for each scenario.

**Table 4:** Key Results from Scenarios

	S.1-Baseline			S.2-Soil productivity			S.3-Green revolution		
	Time period			Time period			Time period		
	1	5	10	1	5	10	1	5	10
**Soil productivity** - Western region *(index)*	1.00	1.00	1.00	1.00	0.96	0.90	1.00	1.14	1.29
**Agricultural inputs** - Total rural use *(index)*	-	-	0.01		-	-	-	0.82	0.35
**Labor** *(MM workers) Food*									
- Western region	2.34	2.26	2.18	2.34	2.49	2.75	2.34	1.15	1.09
- Total rural	9.43	9.15	8.86	9.43	9.98	10.94	9.43	4.88	4.72
*Cash crop* - Western region	1.29	1.35	1.41	1.29	1.17	0.97	1.29	2.26	2.29
- Total rural	4.57	4.79	5.01	4.57	4.14	3.40	4.57	8.22	8.34
*Rural service* - Western region	0.77	0.78	0.78	0.77	0.73	0.67	0.77	0.98	0.98
- Total rural	2.88	2.90	2.93	2.88	2.73	2.48	2.88	3.72	3.69
*Urban* - Service	0.47	0.49	0.50	0.47	0.45	0.40	0.47	0.60	0.62
- Manufacturing	1.81	1.83	1.86	1.81	1.86	1.93	1.81	1.73	1.78
**Migration** *(MM workers)* - Total rural to urban	0.01	0.01	0.01	0.01	0.01	-	0.01	0.02	0.01
**Prices** - Rural food (Western)	0.36	0.38	0.39	0.36	0.43	0.55	0.36	0.20	0.21
- Urban food	0.81	0.78	0.75	0.81	0.88	1.05	0.81	0.41	0.41
- Rural NCPI (Western)	0.86	0.88	0.91	0.86	0.89	0.93	0.86	0.94	0.98
- Urban NCPI	0.85	0.85	0.85	0.85	0.84	0.83	0.85	0.88	0.88
**Real wage** - Rural service (Western)	0.40	0.41	0.43	0.40	0.40	0.39	0.40	0.54	0.58
- Urban service	0.54	0.55	0.55	0.54	0.48	0.36	0.54	0.78	0.77
**Savings** - Gross national rate (%)	11.0	11.2	11.4	11.0	10.3	9.1	11.0	15.1	14.9
**Capital** - Cash crop (Western)	5.00	5.06	5.20	5.00	5.05	5.12	5.00	5.19	5.75
- Rural service (Western)	5.00	4.85	4.73	5.00	4.84	4.68	5.00	4.94	5.10
- Urban service	10.00	9.69	9.38	10.00	9.67	9.20	10.00	9.81	9.80
- Manufacturing	10.00	9.79	9.59	10.00	9.76	9.40	10.00	9.90	10.00
**Government** - Total expenditure	2.09	2.11	2.13	2.09	2.12	2.16	2.09	2.13	2.15
- Tax revenues	1.26	1.32	1.40	1.26	1.26	1.24	1.26	1.81	1.90
**External** - Exports	5.24	5.53	5.84	5.24	5.24	5.07	5.24	7.73	8.46
- Imports	6.10	6.34	6.60	6.10	6.12	6.02	6.10	8.87	8.73
-FDI	0.03	0.03	0.03	0.03	0.03	0.03	0.03	0.02	0.03
**Aid** - Cash ODA	0.84	0.79	0.73	0.84	0.86	0.92	0.84	0.32	0.25
- Green revolution ODA	-	-	-	-	-	-	-	0.80	-
**Total private output** - Real index (year 1 = 100)	100.0	99.9	100.1	100.0	96.8	92.2	100.0	120.1	122.4

*Source:* Authors’ analysis based on model described in text.

*Note:* Prices are in domestic terms relative to a fixed nominal exchange rate and numeraire tradable prices. FDI = foreign direct investment; NCPI = non-food consumer price index; ODA = official development assistance.

### Scenario 1: The Baseline

The baseline scenario shows general stagnation in a low-productivity rural economy. The majority of farm families remain stuck on farms, and labor moves out of food production very slowly over time, with small increases in cash crop labor and otherwise gradual migration to rural services and urban areas. Manufacturing substitutes a growing workforce for its declining capital stock over time. There is almost no modern input use, which is a good general approximation of the historical situation in Uganda.

The rural and urban areas have distinct price dynamics. Food prices vary across rural areas; in period 1 they are 37 percent higher in the Central region than in the Northern region, due to varying initial transport losses, while Western and Eastern region prices are within that range. Food is approximately twice as expensive in the urban compared to rural areas. Over the 10-year period, food prices climb from 3 to 11 percent across rural regions and decline nearly 8 percent in the urban area as the road network improves. The rural and urban non-food CPIs *(NCPI)* are more similar to each other, as services are provided locally without transport costs. The price of rural services increases slightly over time as rural consumption grows, while remaining fairly constant in urban areas. Real wages are presented only for the service sectors because there is cross-sector wage equilibration within each respective rural and urban area. The urban real wage is 36 percent greater than its rural Western counterpart in the first period, with the gap declining to 28 percent by the end of 10 years.

The national savings rate grows modestly over 10 years, from 11.0 percent to 11.4 percent, driven mainly by rural savings. In rural areas a slight majority of investment is directed toward cash crops over services, and is adequate to outweigh depreciation and lead to slow capital accumulation. But even this is not generally enough to cross the threshold for agricultural input use, so only two regions (Eastern and Western) are able to start using even the smallest amount of inputs by Year 10. In the urban areas, local savings are allocated evenly to domestic services and manufacturing, the latter supplemented by FDI. In this scenario, capital depreciation is prevalent across the service and manufacturing sectors.

In the government accounts, 60 percent of the budget is covered by domestic tax revenues, which grow faster than expenditures. The remainder is financed by ODA budget support. External balances mirror the government balance, with a trade deficit equal to 14 percent of total imports. Initial exports are equivalent to 40 percent of GNP, which is higher than Uganda’s actual share, but nonetheless a reference point for the subsequent scenarios. Foreign direct investment is small, reflecting the low return on capital in the country and the low level of investment in the manufacturing sector. The bottom row of 4 shows the lack of overall growth in the real economy over the course of a decade, as an index of real private output.

### Scenario 2: Soil Productivity

This scenario introduces a small amount of annual nutrient variation in the agricultural production functions, adding up to less than 10 percent cumulative soil productivity loss over 10 years. The simulation is again not intended to offer false precision regarding quantitative effects, but it does demonstrate the extent to which a small loss in soil productivity can have considerable negative economy-wide effects, consistent with the findings of Marenya and Barrett (2009) and Matsumoto and Yamano (2009). Compared to the preceding baseline, a marginal decline in agricultural productivity needs to be met by a shift in labor to food crops in order to maintain minimum economy-wide food production. This contributes to cash crop labor decreasing by more than a quarter over the full period. Total farm labor gradually increases, so the rural and urban service sectors also experience labor declines. Only manufacturing absorbs a slightly larger amount of labor than in scenario 1, although rural to urban migration now stops altogether by Year 10.

The other major implication for the economy is significant inflation in food prices, which climb 44 to 56 percent across the four rural regions. By Year 10, urban food prices are also 40 percent higher compared to those of the baseline scenario. The urban real wage drops by more than a third due to the higher food price, even as the rural real wage adjusts only slightly. The final year’s national savings rate is only 9.1 percent.

In the government accounts, tax collections are flat and labor costs increase due mainly to increased service sector wages. By Year 10, the need for cash ODA is considerably higher than in scenario 1, covering nearly 43 percent of the full budget. In this scenario, physical cash crop production drops 20 percent over 10 years. A poverty trap is reflected in a 0.8 percent average annual decline in total private output over the decade.

### Scenario 3: The Green Revolution Package

This scenario introduces the ODA-supported agricultural input package, *greenoda,* with starkly different outcomes compared to the previous two scenarios. The top row of 4 shows how this leads to an increase in the soil productivity parameter, which in turn provides a major boost to overall outcomes. Soil nutrients are replenished by both inputs and higher yields, so soil productivity jumps from 23 to 29 percent across rural regions, with growth rates leveling off once the support package is removed. The boost in staple food productivity frees up labor for cash crops and services in rural areas, while boosting migration into urban areas. 5 presents the scenario’s simulation results for key variables across all 10 years in order to allow a more careful review of the dynamics.

**Table 5:** Key Details for Scenario 3— Green Revolution

	Time period									
	1	2	3	4	5	6	7	8	9	10
**Soil productivity** - Western region *(index)*	1.00	0.99	0.98	1.07	1.14	1.19	1.23	1.26	1.28	1.29
**Agricultural inputs** - Total rural use *(index)*	-	-	1.20	1.00	0.82	0.68	0.55	0.22	0.28	0.35
**Labor** *(MM workers)* *Food* - Western	2.34	2.37	1.37	1.22	1.15	1.10	1.09	1.21	1.15	1.09
- Total rural	9.43	9.55	5.77	5.17	4.88	4.72	4.67	5.17	4.95	4.72
*Cash crop* - Western	1.29	1.26	2.07	2.20	2.26	2.29	2.30	2.20	2.24	2.29
- Total rural	4.57	4.48	7.51	8.00	8.22	8.36	8.39	7.98	8.15	8.34
*Rural service* - Western	0.77	0.76	0.95	0.97	0.98	0.99	0.99	0.96	0.97	0.98
- Total rural	2.88	2.85	3.58	3.68	3.72	3.74	3.73	3.63	3.66	3.69
*Urban* - Service	0.47	0.47	0.58	0.59	0.60	0.61	0.61	0.60	0.61	0.62
- Manufacturing	1.81	1.82	1.72	1.72	1.73	1.74	1.75	1.77	1.78	1.78
**Prices** - Rural food (Western)	0.36	0.38	0.22	0.20	0.20	0.20	0.20	0.22	0.22	0.21
- Urban food	0.81	0.82	0.47	0.43	0.41	0.40	0.40	0.44	0.42	0.41
- Rural NCPI (Western)	0.86	0.86	0.90	0.93	0.94	0.96	0.96	0.95	0.97	0.98
- Urban NCPI	0.85	0.85	0.88	0.88	0.88	0.88	0.88	0.88	0.88	0.88
**Real wage** - Rural service (Western)	0.40	0.40	0.50	0.53	0.54	0.55	0.56	0.55	0.56	0.58
- Urban service	0.54	0.53	0.75	0.77	0.78	0.78	0.78	0.76	0.77	0.77
**Savings** - Gross national rate (%)	11.0	10.8	14.4	14.9	15.1	15.2	15.1	14.6	14.7	14.9
**Government** - Total expenditure	2.09	2.10	2.10	2.12	2.13	2.14	2.14	2.14	2.14	2.15
- Tax revenues	1.26	1.26	1.63	1.74	1.81	1.85	1.88	1.80	1.85	1.90
**ODA** - Cash ODA	0.84	0.84	0.47	0.38	0.32	0.28	0.27	0.34	0.30	0.25
- Green revolution ODA	-	-	1.20	1.00	0.80	0.60	0.40	-	-
**Total private output** - Real index (year 1 = 100)	100.0	99.2	114.9	118.3	120.1	121.3	121.9	119.0	120.5	122.4

*Source:* Authors’ analysis based on model described in text.

*Note:* Prices are in domestic terms relative to a fixed nominal exchange rate and numeraire tradable prices. FDI = foreign direct investment; NCPI = non-food consumer price index; ODA = official development assistance.

The green revolution ODA package, summed across rural regions in tables 4 and 5, begins in Year 3 and gradually declines through to Year 7. Then it is withdrawn entirely in Year 8, prompting a temporary increase in staple food labor, but the downward trend then resumes as labor continues to move into all the other higher-paying sectors. By that point farmers are able to generate enough capital in cash crops to initiate a self-sustaining and growing agricultural input market in all rural areas. In other words, under reasonable parameters a temporary boost in targeted ODA for agriculture leads to a permanent boost in agricultural productivity.

Price dynamics form an important element of this scenario. By Year 7, food prices drop by at least 43 percent in all rural regions, and by 51 percent in the urban area. In the total consumption basket, this generally outweighs the non-food CPI increases of 5 to 15 percent across rural areas over the decade, while the urban non-food CPI increases by only 4 percent. By Year 10, real wages in rural services increase by 26 to 46 percent, and in urban services by 43 percent. These price trends demonstrate one way in which a targeted ODA support package might avoid traditional Dutch disease dynamics.

Meanwhile, savings receives a boost from the increase in disposable income, leading to a savings rate that grows to 14.9 percent by Year 10. Underlying real output grows 22.4 percent over the same horizon, for a compound growth rate of 2.0 percent per year. Major expansion of the exported cash crop sector drives a considerable share of the overall growth, again counter to Dutch disease-type concerns about aid hindering growth in key tradable sectors. Over the final three years, overall real growth rates in private output are -2.4 percent, 1.3 percent, and then 1.5 percent, respectively. By the end of the scenario, the underlying overall capital stocks across all sectors are able to halt, and in most instances reverse, declines experienced in the pre-green revolution years.

A major boost to tax revenues leads to rapid progress on the government balances. In the final year of the green revolution package, Year 7, budget support ODA (i.e., excluding the ODA for agricultural inputs) is less than 12 percent of government expenditures and only 1.6 percent of GNP. After a downward adjustment in Year 8, the tax revenue growth trajectory reinitiates in Year 9, and by Year 10 the government budget is again approaching full domestic financing.

## 4 Discussion

**Figure 1:**
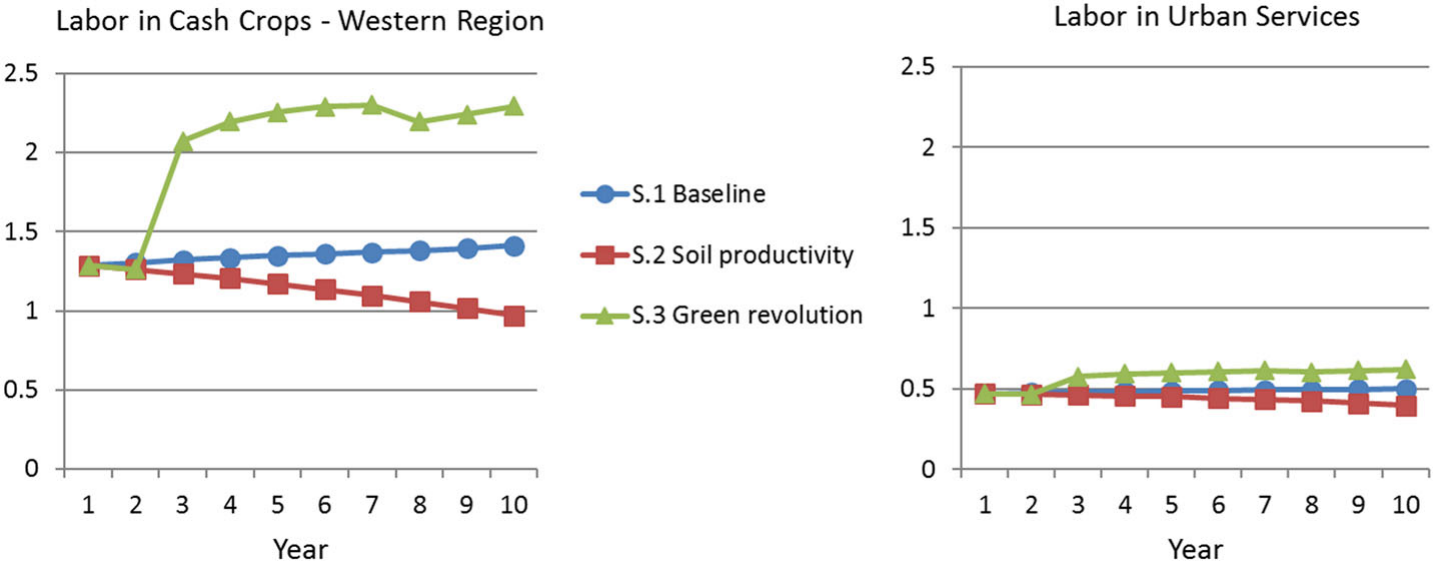
Sectoral Labor Shifts *Source:* Authors’ analysis based on model described in text. *Note:* S.1 = scenario 1; S.2 = scenario 2; S.3 = scenario 3.

The simulations illustrate some of the dynamics that can be evaluated by considering specific pathways for targeting aid. Figures.1 through 6 compare the paths of some key variables across scenarios. The left panel of fig. 1 shows labor in cash crops in the indicative Western region. It demonstrates that the baseline (scenario 1) has relatively stable labor in cash crops. The introduction of a soil productivity loss function instigates a clear decline of labor in the sector, because labor must shift back to food production in order to meet the country’s minimum needs. On the other hand, the green revolution scenario shows a rapid and sustained increase in tradable cash crop labor.

The right panel of fig. 1 presents the trends for labor in urban services. The baseline scenario shows a very gradual increase in the sector, but the soil nutrient scenario experiences a decline in urban (and rural) service labor due to the requirements for food production. Again, the green revolution prompts a 32 percent increase in urban service employment as its capital stock is bolstered through increased savings and higher farm productivity leads to a decline in the total number of farmworkers.

**Figure 2:**
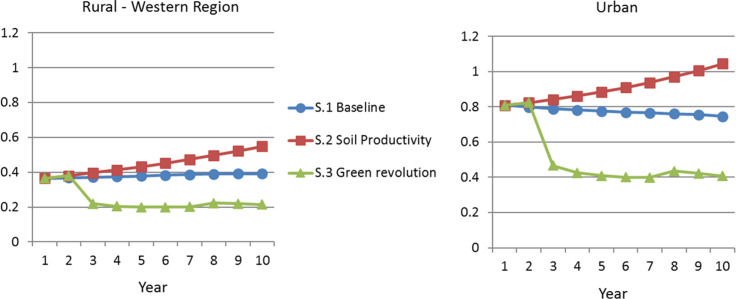
Food Price *Source*: Authors’ analysis based on model described in text. *Note*: S.1 = scenario 1; S.2 = scenario 2; S.3 = scenario 3.

Figure 2 shows trajectories for rural and urban food prices. A clear pattern emerges here too. The scenario 1 baseline sees general food price stability, but the incorporation of soil nutrient loss initiates a path of food price inflation in all regions, while the green revolution scenario sees the opposite effect. Part of this is driven by the model’s structural imperative of meeting *phi,* the fixed underlying demand for food. The green revolution scenario’s dual effects of labor moving from nontradable staple foods to tradable cash crops while food prices drop indicates progress through labor’s movement into a higher-value sector while also generating higher real incomes.

**Figure 3:**
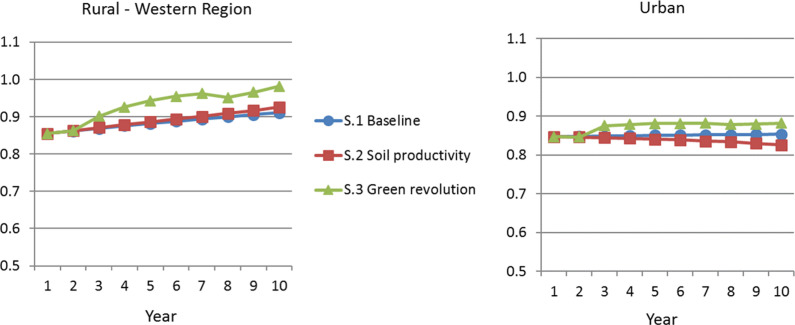
Non-Food Consumer Price Index *Source*: Authors’ analysis based on model described in text. *Note:* S.1 = scenario 1; S.2 = scenario 2; S.3 = scenario 3.

Figure 3 shows the non-food CPI trends, with much smaller relative price fluctuations. Across all scenarios, the urban non-food prices are relatively stable. Under the green revolution scenario, rural nonfood prices in three regions (Central, Eastern, Western) climb 7 to 9 percent higher over the decade than under the baseline scenario, due to income growth, while the Northern region’s prices climb only slightly less than under the baseline.

The implications for real exchange rate dynamics are informative. Once both food and non-food prices are taken into account, the agricultural intensification scenario shows an overall trend whereby imported agricultural inputs lead to a major expansion of cash crop exports alongside strong deflationary consequences for the rural populations, even while urban prices remain fairly constant.^7^ The balance of price effects is determined by food’s share in the consumption basket—which is large in this case. This occurs alongside an 84 percent expansion in cash crop exports. The ODA-related discussions of Dutch disease might need to introduce new jargon for the relative price effects caused by ODA targeted to staple crop productivity—”agro-ease”?

**Figure 4:**
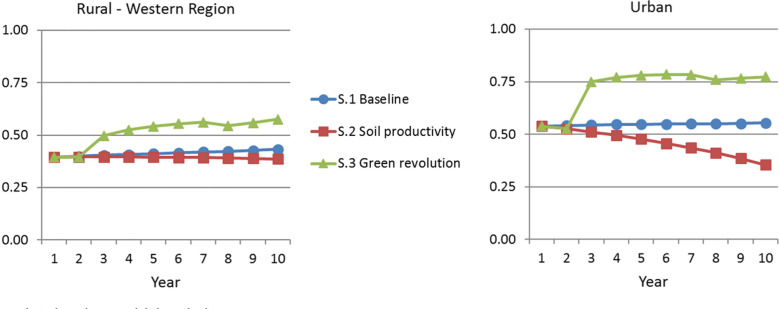
Real Wage *Source:* Authors’ analysis based on model described in text. *Note:* S.1 = scenario 1; S.2 = scenario 2; S.3 = scenario 3.

Figure 4 highlights another major outcome of interest by comparing real wages. Again, there is a stark distinction between scenarios defined by soil productivity loss versus smallholder productivity gains, including major differences in the urban sectors. In scenarios 1 and 2, rural real wages change very little over the decade, while in scenario 3, they increase by 26 to 46 percent across regions. The differences in urban outcomes are much starker. Under conditions of unaddressed soil productivity decline, real urban wages drop 34 percent. This compares to the green revolution strategy, wherein urban wages grow 43 percent. A key implication is that rural staple productivity trends can have large effects on urban living standards, independent of any direct interventions in the urban sector.

**Figure 5:**
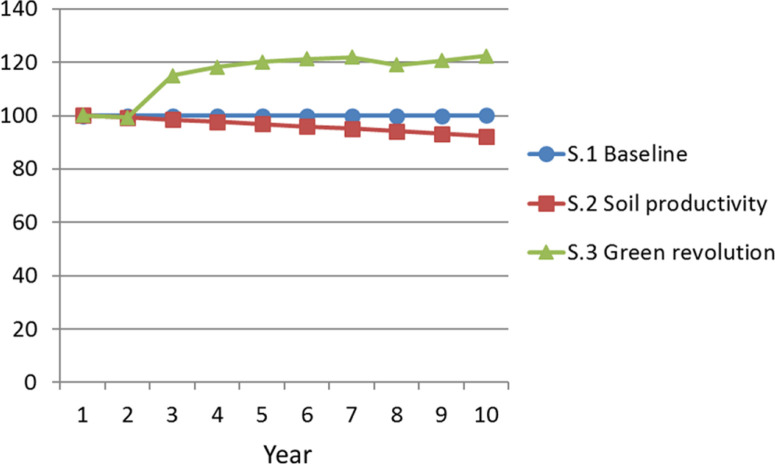
Total Private Output - Real Index (year 1 = 100) *Source*: Authors’ analysis based on model described in text. *Note:* S.1 = scenario 1; S.2 = scenario 2; S.3 = scenario 3.

Figure 5 compares trajectories for real growth in (constant price) total private output, with Year 1 set to an index value of 100. The baseline scenario shows overall output stagnation, and the soil productivity that is affected by transport costs, but it does not play a major role in determining non-food price indexes in an economy with no intermediate goods. scenario shows a gradual long-term decline. Meanwhile, the green revolution simulation again shows a distinctly higher growth path. The jump in cash crop production drives the economy to a much higher new level of output as of Year 3. The economy continues to grow under the period of targeted agricultural support over Years 4 through 7, then experiences a downward adjustment in Year 8 before re-initiating a new positive growth trend.

**Figure 6:**
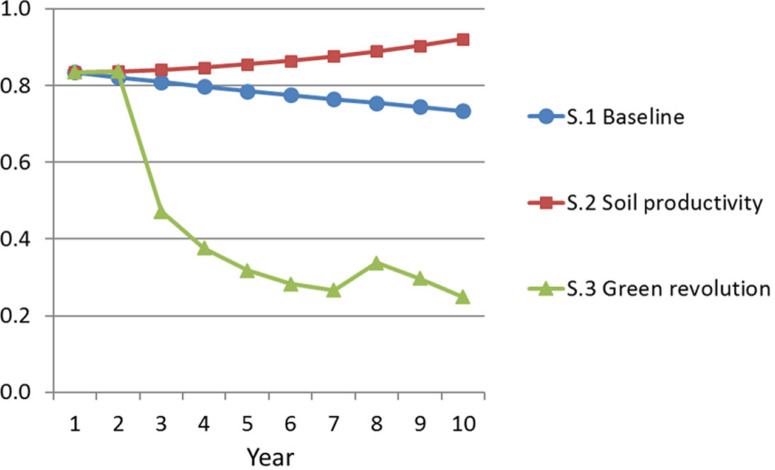
Budget Support ODA *Source:* Authors’ analysis based on model described in text. *Note:* S.1 = scenario 1; S.2 = scenario 2; S.3 = scenario 3.

Figure 6 presents perhaps the most dramatic results from the vantage point of considering potential consequences of different types of aid. The three lines indicate paths of aid for budget support, *CASHODA,* under the three respective scenarios. The contrasting directional dynamics are clear. The baseline scenario charts a long and slow decline in cash ODA as the economy experiences very gradual nominal growth. A simple extrapolation of the trend suggests it would take another several decades for the country to graduate from the need for budget support. Meanwhile the poverty trap dynamics in the soil productivity scenario require ongoing *expansion* of budget support ODA in order to fill the government’s growing primary deficit.

The green revolution strategy follows an entirely different pattern. Under scenario 3, the decline in required budget support begins immediately in Year 3, due to the major growth effects the same year, when complemented by the introduction of considerable agricultural input support. The value of “total” (budget plus input support) ODA in Year 3 would be greater than the value under budget support alone, noting that the addition of two different types of ODA should be interpreted with caution. Then, as of Year 4, both budget support and *greenoda* are declining. After the final installment of agricultural input support in Year 7, there is a one-time increase in Year 8, when budget support increases to a level slightly higher than in Year 5, before resuming a downward trend again thanks to the cumulative growth effects.

One potential interpretation of fig. 6 is that modest soil nutrient loss in rural subsistence-dominated economies can drive the need for budget support’s persistence, while nutrient supplementation can instead help drive its decline. In light of ongoing debates in the literature, we caution against interpreting this result to imply that aid-backed input support packages will automatically generate the latter outcomes. A growing evidence base is taking shape regarding the strengths and weaknesses of different input support programs under different contexts. Nonetheless, the result does illustrate a set of potential dynamics linked to an efficacious and time-limited input support program.

## 5 Conclusion

This paper presents a macroeconomic model to enable insights regarding the channels through which basic issues of soil nutrients and targeted aid for agricultural input support could promote economic growth in Africa. The model makes a number of simplifying assumptions in order to focus on key issues of interest under three stylized scenarios. The most important dynamics pertain to the equilibrium impact of introducing a broadly scaled small-holder modern agricultural input support strategy, including complementary goods such as fertilizer and modern variety seeds. The model highlights the significant potential macroeconomic consequences of soil nutrient flows, both negative and positive, with implications for understanding the possible persistence of—or escape from—potential poverty traps in rural Africa.

Under plausible parameters, the model illustrates that a green revolution strategy could allocate a temporary boost in targeted ODA to generate permanent productivity, income, and welfare effects in both rural and urban areas. The results highlight the importance of unpacking the different potential pathways and outcomes produced by different aims and purposes of allocating aid. It also underscores the risk of ignoring agriculture’s potential role in stimulating improved living standards in other sectors, including urban sectors.

The model’s empirical results demonstrate directions and pathways rather than precise point estimates. The model itself offers considerable range for scenario-building, parameterizations, and additional refinements that could enhance its ability to generate analytical insights around specific questions. Potential offshoot analyses could include, first, more nuanced labor mobility functions. While labor in the model is parameterized to migrate gradually between the rural and urban sectors, rapid mobility between the food and cash crop sectors is fundamental to the dynamics and to the positive general outcomes in the green revolution scenario. In reality, most farmers have a high degree of mobility between these two sectors in making planting choices on their own farms, but switching costs are likely nonzero, and the literature on farmer adoption patterns suggests that the instantaneous assumption is a simplification.

Second, the production functions could be refined in a number of ways. For example, the soil nutrient flows could be calibrated to specific regions, and more explicitly linked to production volumes and plant residues. The model also represents agricultural inputs as purely labor-saving, which is not necessarily the case because, for example, the introduction of fertilizer can require additional weeding and land management. The manufacturing sector could also incorporate more domestic and external factors affecting growth and learning-by-doing. The production functions could explore more sophisticated human capital structure or constant elasticity of transformation technologies where the additional modeling complexities would help illuminate key topics of interest.

Third, the model excludes demographic effects, which are important due to shrinking land/labor ratios in many African countries and because investments in health, education, and agricultural productivity are all likely to lead to increases in life expectancy and decreases in fertility rates. The government sector equations could be explored to examine these issues. Fourth, models could add a natural resource sector, in line with recent discoveries in countries like Uganda. Fifth, alternatives to the iceberg model could be considered for agricultural transport assumptions. Incorporating any of these issues would further augment the model’s ability to inform macroeconomic analysis of relevant issues across African economies.

## Footnotes

1 The core Lofgren, Harris, and Robinson model has been applied to many countries—including Dorosh, El-Said, and Lofgren’s (2002) application to Uganda, which finds that positive agricultural productivity shocks provide less of a rural welfare boost than investments to decrease marketing margins.

2 The non-agricultural productive sectors—urban manufacturing, urban services, and rural services—also take a simple Cobb-Douglas form.

3 This shorthand calculation for complex underlying nutrient flows facilitates solvability in the numerical model.

4 In the simulations presented here, the *phi* value of 0.55 is set as a reasonable first approximation for the broader dynamics of the model. Future research could provide more precision on staple crop volume needs by region. Advances in soil nutrient mapping could also help develop location-specific and crop-specific farm productivity thresholds.

5 Future research could explore adding further complexity to the model by including a domestic transport sector that incorporates product-specific import transport costs.

6 The government module could be easily modified to add any number of service sectors or ministry-type accounts, addressing urban investment costs, rural investment costs, and recurrent costs. The simulations presented in this paper include four illustrative sectors—health, education, infrastructure, and public administration—in addition to road building and maintenance. Labor costs are determined by staff numbers and the relative wage, which is in turn determined by service sector wages. Imported commodity inputs can also be easily specified to a particular year.

7 Road building helps mitigate inflation among staple food as a primary consumption good
