# Supplementary material for: Agriculture, Aid, and Economic Growth in Africa
Source: World Bank Econ Rev. 2018 Jun 13;33(1):1–20. doi: 10.1093/wber/lhx029 (PMC7797631; doi:10.1093/wber/lhx029)
Supplement: Supplementary file 1 [file WBER-33-01-001-s001.pdf]

Supplementary Material

Agriculture, Aid, and Economic Growth in Africa

John W. McArthur and Jeffrey D. Sachs

Supplementary Online Appendix S1: Model Equation Listing

BLOCK 1: AGRICULTURAL PRODUCTION FUNCTIONS

$$F_{i,t} = S_{i,t} * thetaf_i * land_i * (1 + INPUT_{i,t}) * LF_{i,t}^{alphaf} * kf_i^{betaf} \quad \text{(EQ.1.1)}$$

$$VF_{i,t} = F_{i,t} * PFR_{i,t} \quad \text{(EQ.1.2)}$$

$$CC_{i,t} = S_{i,t} * thetacc_i * land_i * (1 + INPUT_{i,t}) * LCC_{i,t}^{alphacc} * (kcscale * KCC_{i,t})^{betacc} \quad \text{(EQ.1.3)}$$

$$VCC_{i,t} = CC_{i,t} * PCCR_{i,t} \quad \text{(EQ.1.4)}$$

$$S_{i,t} = \frac{uppersoil_i}{1 + rho * EXPNUT_{i,t}} \quad \text{(EQ.1.5)}$$

$$EXPNUT_{i,t} = e^{-NUTSUM_{i,t}} \quad \text{(EQ.1.6)}$$

$$NUTSUM_{i,t+1} = NUTSUM_{i,t} + NETIN_{i,t} \quad \text{(EQ.1.7)}$$

$$NUTSUM_{i,t0} = nutrient0_i \quad \text{(EQ.1.8)}$$

$$NETIN_{i,t} = INPUT_{i,t} - nlossrate \quad \text{(EQ.1.9)}$$

$$INPUT_{i,t} = min(1, max(0, (kcscale * (KCC_{i,t} - ((1 + khurdle) * kcc0_i)))) + greenoda_t) \quad \text{(EQ.1.10)}$$

BLOCK 2: NONAGRICULTURAL PRODUCTION FUNCTIONS

$$M_t = thetam_t * LM_t^{alpham} * (kmscale * KM_t)^{1-alpham} \quad \text{(EQ.2.1)}$$

$$SU_t = thetasu_t * LSU_t^{alphas} * (ksscale * KSU_t)^{1-alphas} \quad \text{(EQ.2.2)}$$

$$VSU_t = SU_t * PSU_t \quad \text{(EQ.2.3)}$$

$$SR_{i,t} = thetasr_i * LSR_{i,t}^{alphas} * (ksscale * KSR_{i,t})^{1-alphas} \quad \text{(EQ.2.4)}$$

$$VSR_{i,t} = SR_{i,t} * PSR_{i,t} \quad \text{(EQ.2.5)}$$

BLOCK 3: FOOD MARKET CLEARANCE

$$FKAMP_t = phi * LU_t \quad \text{(EQ.3.1)}$$

$$FKAMP_t = \sum_i FSURP_{i,t} * (1 - TLOSS_{i,t}) \quad \text{(EQ.3.2)}$$

$$FSURP_{i,t} = F_{i,t} - phi * LR_{i,t} \quad \text{(EQ.3.3)}$$

BLOCK 4: TRANSPORT COSTS & PRICES

$$TLOSS_{i,t} = \frac{tlossfirst0_i}{kroadscale * KROAD_{i,t}} \quad \text{(EQ.4.1)}$$

$$PFR_{i,t} = PFU_t * (1 - TLOSS_{i,t}) \quad \text{(EQ.4.2)}$$

$$PCCR_{i,t} = 1 - TLOSS_{i,t} \quad \text{(EQ.4.3)}$$

$$CPINFU_t = PSU_t * gammas + pimpu_t * (1 - gammas) \quad \text{(EQ.4.4)}$$

$$CPINFR_{i,t} = PSR_{i,t} * gammas + pimpr_t * (1 - gammas) \quad \text{(EQ.4.5)}$$

BLOCK 5: LABOR INCOME BY SECTOR

$$WMAN_t = thetam_t * alpham * (kmscale * KM_t / LM_t)^{1-alpham} \quad \text{(EQ.5.1)}$$

$$WSU_t = PSU_t * thetasu_t * alphas * (ksscale * KSU_t / LSU_t)^{1-alphas} \quad \text{(EQ.5.2)}$$

$$WSR_{i,t} = PSR_{i,t} * alphas * thetasr_i * (ksscale * KSR_{i,t} / LSR_{i,t})^{1-alphas} \quad \text{(EQ.5.3)}$$

$$MPF_{i,t} = \frac{PFR_{i,t} * F_{i,t} * alphaf}{LF_{i,t}} \quad \text{(EQ.5.4)}$$

$$MPCC_{i,t} = \frac{PCCR_{i,t} * CC_{i,t} * alphacc}{LCC_{i,t}} \quad \text{(EQ.5.5)}$$

$$WGR_{i,t} = pwageprem * WSR_{i,t} \quad \text{(EQ.5.6)}$$

$$WGU_t = pwageprem * WSU_t \quad \text{(EQ.5.7)}$$

$$MPCC_{i,t} = MPF_{i,t} \quad \text{(EQ.5.8)}$$

$$RWMAN_t = \frac{WMAN_t - phi * PFU_t}{CPINFU_t} \quad \text{(EQ.5.9)}$$

$$RWSU_t = \frac{WSU_t - phi * PFU_t}{CPINFU_t} \quad \text{(EQ.5.10)}$$

$$RWSR_{i,t} = \frac{WSR_{i,t} - phi * PFR_{i,t}}{CPINFR_{i,t}} \quad \text{(EQ.5.11)}$$

$$YFARM_{i,t} = \frac{VF_{i,t} + VCC_{i,t} - phi * PFR_{i,t} * LFARM_{i,t} - (INPUT_{i,t} - greenoda_t)}{CPINFR_{i,t} * LFARM_{i,t}} \quad \text{(EQ.5.12)}$$

$$RWGOVU_t = \frac{WGU_t - phi * PFU_t}{CPINFU_t} \quad \text{(EQ.5.13)}$$

$$RWGOVR_{i,t} = \frac{WGR_{i,t} - phi * PFR_{i,t}}{CPINFR_{i,t}} \quad \text{(EQ.5.14)}$$

BLOCK 6: LABOR MIGRATION AND MARKET EQUILIBRIUM

$$RWSU_t = RWMAN_t \quad \text{(EQ.6.1)}$$

$$RWSR_{i,t} = YFARM_{i,t} \quad \text{(EQ.6.2)}$$

$$MIGRATE_{i,t} = migtheta * (RWSU_t - RWSR_{i,t}) \quad \text{(EQ.6.3)}$$

$$LR_{i,t+1} = LR_{i,t} - MIGRATE_{i,t} \quad \text{(EQ.6.4)}$$

$$LR_{i,t0} = lr0_i \quad \text{(EQ.6.5)}$$

$$LR_{i,t} = LFARM_{i,t} + LSR_{i,t} + LGR_{i,t} \quad \text{(EQ.6.6)}$$

$$LFARM_{i,t} = LF_{i,t} + LCC_{i,t} \quad \text{(EQ.6.7)}$$

$$LU_t = LM_t + LSU_t + LGU_t \quad \text{(EQ.6.8)}$$

$$LU_t = \sum_i pop_i - \sum_i LR_{i,t} \quad \text{(EQ.6.9)}$$

$$LG_t = \sum_i LGR_{i,t} + LGU_t \quad \text{(EQ.6.10)}$$

$$LTOT_t = \sum_i LR_{i,t} + LU_t \quad \text{(EQ.6.11)}$$

BLOCK 7: DISPOSABLE INCOME, TAXATION, CONSUMPTION, AND SAVING

$$YU_t = (VSU_t + M_t + GOVWU_t - phi^* PFU_t{}^* LU_t)^* (1 - taxr) \quad \text{(EQ.7.1)}$$

$$\begin{aligned} YR_{i,t} = {} & (VF_{i,t} + VCC_{i,t} + VSR_{i,t} + GOVWR_{i,t} - (INPUT_{i,t} - greenoda_t) \\ & - phi^* PFR_{i,t}{}^* LR_{i,t})^* (1 - taxr) \end{aligned} \quad \text{(EQ.7.2)}$$

$$YRTOT_t = \sum_i YR_{i,t} \quad \text{(EQ.7.3)}$$

$$YDIS_t = YU_t + \sum_i YR_{i,t} \quad \text{(EQ.7.4)}$$

$$SAVU_t = max(0, (YU_t - (CPINFU_t{}^* cmin^* LU_t))^* savurb) \quad \text{(EQ.7.5)}$$

$$SAVR_{i,t} = max(0, (YR_{i,t} - (CPINFR_{i,t}{}^* cmin^* LR_{i,t}))^* savrur) \quad \text{(EQ.7.6)}$$

$$SAVTOT_t = SAVU_t + \sum_i SAVR_{i,t} \quad \text{(EQ.7.7)}$$

$$CU_t = YU_t - SAVU_t \quad \text{(EQ.7.8)}$$

$$CR_{i,t} = YR_{i,t} - SAVR_{i,t} \quad \text{(EQ.7.9)}$$

$$SU_t = \frac{gammas^* CU_t}{PSU_t} \quad \text{(EQ.7.10)}$$

$$SR_{i,t} = \frac{gammas^* CR_{i,t}}{PSR_{i,t}} \quad \text{(EQ.7.11)}$$

BLOCK 8: TRADE BALANCE

$$IMPUC_t = (1 - gammas)^* CU_t \quad \text{(EQ.8.1)}$$

$$IMPRC_{i,t} = (1 - gammas)^* CR_{i,t} \quad \text{(EQ.8.2)}$$

$$IMPUI_t = SAVU_t + FDI_t \quad \text{(EQ.8.3)}$$

$$IMPRI_{i,t} = SAVR_{i,t} \quad \text{(EQ.8.4)}$$

$$EXPORT_t = \sum_i (CC_{i,t}{}^* (1 - TLOSS_{i,t})) + M_t \quad \text{(EQ.8.5)}$$

$$\begin{aligned} IMPORT_t = {} & \sum_i INPUT_{i,t} + \sum_i IMPRC_{i,t} + IMPUC_t + \sum_i IMPRI_{i,t} + IMPUI_t \\ & + PUBINV_t + ROADINV_t + TOTIMPG_t \end{aligned} \quad \text{(EQ.8.6)}$$

$$TB_t = EXPORT_t - IMPORT_t \quad \text{(EQ.8.7)}$$

BLOCK 9: INVESTMENT AND CAPITAL ACCUMULATION

$$FDI_t = max(0, fdimult^* (R_t - rworld)) \quad \text{(EQ.9.1)}$$

$$R_t = \frac{(1 - alpham)^* M_t}{KM_t} \quad \text{(EQ.9.2)}$$

$$KCC_{i,t+1} = KCC_{i,t}{}^* (1 - depcc) + rsavcc^* SAVR_{i,t} \quad \text{(EQ.9.3)}$$

$$KM_{t+1} = KM_t{}^* (1 - dep) + FDI_t + usavm^* SAVU_t \quad \text{(EQ.9.4)}$$

$$KCC_{i,t0} = kcc0_i \quad \text{(EQ.9.5)}$$

$$KM_{t0} = km0 \quad \text{(EQ.9.6)}$$

$$KSU_{t0} = ksu0 \quad \text{(EQ.9.7)}$$

$$KSR_{i,t0} = ksr0_i \quad \text{(EQ.9.8)}$$

$$KSU_{t+1} = KSU_t{}^* (1 - dep) + (1 - usavm)^* SAVU_t \quad \text{(EQ.9.9)}$$

$$KSR_{i,t+1} = KSR_{i,t}{}^* (1 - dep) + (1 - rsavcc)^* SAVR_{i,t} \quad \text{(EQ.9.10)}$$

BLOCK 10: PUBLIC SECTOR

a) Public Balances

$$TAX_t = (VSU_t + M_t + GOVWU_t - phi^* PFU_t{}^* LU_t)^* taxr + \sum\nolimits_i (VF_{i,t} + VCC_{i,t} + VSR_{i,t} + GOVWR_{i,t} - (INPUT_{i,t} - greenoda_t) - phi^* PFR_{i,t}{}^* LR_{i,t})^* taxr \quad \text{(EQ.10.1)}$$

$$TOTEXP_t = TAX_t + CASHODA_t \quad \text{(EQ.10.2)}$$

$$CODA = \sum\nolimits_t CASHODA_t \quad \text{(EQ.10.3)}$$

$$TOTEXP_t = \sum\nolimits_p PEXP_{p,t} + ROADCOST_t \quad \text{(EQ.10.4)}$$

$$PEXP_{p,t} = invu_{p,t} + \sum\nolimits_i invr_{p,i,t} + RECCOST_{p,t} \quad \text{(EQ.10.5)}$$

$$PUBINV_t = \sum\nolimits_p invu_{p,t} + \sum\nolimits_p \sum\nolimits_i invr_{p,i,t} \quad \text{(EQ.10.6)}$$

b) Roads

$$KROAD_{i,t+1} = KROAD_{i,t}{}^* (1 - dep) + invroad_{i,t} \quad \text{(EQ.10.7)}$$

$$KROAD_{i,t0} = road0_i \quad \text{(EQ.10.8)}$$

$$LROAD_{i,t} = invroad_{i,t}{}^* klroad_i + maintlabor^* KROAD_{i,t} \quad \text{(EQ.10.9)}$$

$$ROADWAGE_{i,t} = LROAD_{i,t}{}^* WGR_{i,t} \quad \text{(EQ.10.10)}$$

$$ROADINV_t = \sum\nolimits_i invroad_{i,t} \quad \text{(EQ.10.11)}$$

$$ROADCOST_t = ROADINV_t + \sum\nolimits_i ROADWAGE_{i,t} \quad \text{(EQ.10.12)}$$

c) Other Public Capital Equations

$$KPU_{p,t+1} = KPU_{p,t} * (1 - dep) + invu_{p,t} \quad \text{(EQ.10.13)}$$

$$KPR_{p,i,t+1} = KPR_{p,i,t} * (1 - dep) + invr_{p,i,t} \quad \text{(EQ.10.14)}$$

$$KPU_{p,t0} = kpu0_p \quad \text{(EQ.10.15)}$$

$$KPR_{p,i,t0} = kpr0_{i,p} \quad \text{(EQ.10.16)}$$

d) Public Service Recurrent Expenditures

$$RECCOST_{p,t} = \sum_{reg} commod_{p,reg,t} + LABCOST_{p,t} \quad \text{(EQ.10.17)}$$

$$LABCOST_{p,t} = lpu_{p,t} * WGU_t + \sum_i (lpr_{p,i,t} * WGR_{i,t}) \quad \text{(EQ.10.18)}$$

$$LGU_t = \sum_p lpu_{p,t} \quad \text{(EQ.10.19)}$$

$$LGR_{i,t} = \sum_p lpr_{p,i,t} + LROAD_{i,t} \quad \text{(EQ.10.20)}$$

$$GOVWU_t = WGU_t * LGU_t \quad \text{(EQ.10.21)}$$

$$GOVWR_{i,t} = WGR_{i,t} * LGR_{i,t} \quad \text{(EQ.10.22)}$$

$$GOVWRUR_t = \sum_i GOVWR_{i,t} \quad \text{(EQ.10.23)}$$

$$GWBILL_t = GOVWU_t * GOVWRUR_t \quad \text{(EQ.10.24)}$$

e) Public Service Import Content

$$TOTIMPG_t = \sum_p IMPG_{p,t} \quad \text{(EQ.10.25)}$$

$$IMPG_{p,t} = \sum_{reg} (impcontc_p * commod_{p,reg,t}) \quad \text{(EQ.10.26)}$$

BLOCK 11: ECONOMIC AGGREGATES

$$FTOT_t = \sum_i F_{i,t} \quad \text{(EQ.11.1)}$$

$$VFTOT_t = \sum_i VF_{i,t} \quad \text{(EQ.11.2)}$$

$$VCCTOT_t = \sum_i VCC_{i,t} \quad \text{(EQ.11.3)}$$

$$VSRTOT_t = \sum_i VSR_{i,t} \quad \text{(EQ.11.4)}$$

$$GNPWF_t = VSU_t + M_t + VFTOT_t + VCCTOT_t + VSRTOT_t + GWBILL_t \quad \text{(EQ.11.5)}$$

$$SAVGNPWF_t = \frac{SAVTOT_t}{GNPWF_t} \quad \text{(EQ.11.6)}$$

Supplementary Online Appendix S2: List of Variables in Model

Positive variables:

$CC_{i,t}$	Cash crop output
$CPINFU_t$	Nonfood consumer price index urban
$CPINFR_{i,t}$	Nonfood consumer price index rural
$CR_{i,t}$	Rural consumption
CU_t	Urban consumption
$EXPNUT_{i,t}$	Exponential of nutrient stock
$EXPORT_t$	Total exports
$EXTRACT_{i,t}$	Crop extraction of nutrients
$F_{i,t}$	Food output
FDI_t	Foreign direct investment
$FSURP_{i,t}$	Food surplus by region
$FTOT_t$	Total food production
GNP_t	National output value
$GOVWR_{i,t}$	Rural government wage bill in region *i*
$GOVWRUR_t$	Total rural government wage bill
$GOVWU_t$	Urban government wage bill
$GWBILL_t$	Total government wage bill
$IMPORT_t$	Total imports
$INPUT_{i,t}$	Modern agricultural input use
$KCC_{i,t}$	Capital in cash crop production
KM_t	Capital in manufacturing
$KPR_{p,i,t}$	Rural capital stock in sector in period
$KPU_{p,t}$	Urban capital stock in sector in period
$KROAD_{i,t}$	Road length
$KSR_{i,t}$	Capital in rural service
KSU_t	Capital in urban service
$LABCOST_{p,t}$	Public labor force cost in sector *p*
$LCC_{i,t}$	Labor in cash crop production
$LF_{i,t}$	Labor in food production
$LFARM_{i,t}$	Farm labor
LG_t	Total government labor
$LGR_{i,t}$	Rural government labor
LGU_t	Urban government labor
LM_t	Labor in manufacturing
$LR_{i,t}$	Labor in rural area
$LROAD_{i,t}$	Rural road building labor
$LSR_{i,t}$	Rural services labor
LSU_t	Urban services labor
LU_t	Labor in urban sector
M_t	Manufacturing output
$MPCC_{i,t}$	Marginal product of labor in cash crop production
$MPF_{i,t}$	Marginal product of labor in food production
$PCCR_{i,t}$	Price of cash crop in rural region
$PFR_{i,t}$	Price of food in rural region
PFU_t	Price of food in urban region
$PSR_{i,t}$	Price of services in rural region
PSU_t	Price of services in urban region

R_t	Marginal product of capital in manufacturing
$RWSR_{i,t}$	Real wage in rural services net of food
$RWSU_t$	Real wage in urban services net of food
$S_{i,t}$	Soil productivity factor
$SR_{i,t}$	Rural services output
SU_t	Urban services output
$TLOSS_{i,t}$	Transport losses
$VCC_{i,t}$	Value added of cash crop production in region
$VCCTOT_t$	Value added of total cash crop production
$VF_{i,t}$	Value of food production in region
$VFTOT_t$	Total value of food production
$VSR_{i,t}$	Value of rural service production
$VSRTOT_t$	Total value of rural service production
VSU_t	Value of urban service production
$WCC_{i,t}$	Cash crop wage
$WGR_{i,t}$	Rural government salary
WGU_t	Urban government salary
$WMAN_t$	Manufacturing wage
$WSR_{i,t}$	Rural service wage
WSU_t	Urban service wage
$YFARM_{i,t}$	Real farm income
$YR_{i,t}$	Rural income in region
$YRTOT_t$	Total rural income

Free variables:

$CASHODA_t$	Cash ODA (non-agricultural budget support)
$CODA_t$	Cumulative cash ODA
$DISYRU_{i,t}$	Rural income in excess of minimum consumption (*cmin*)
$DISYUR_t$	Urban income in excess of minimum consumption (*cmin*)
$FKAMP_t$	Food need in Kampala
$GNPWF_t$	GNP with food
$IMPG_{p,t}$	Import content in public service sector expenditures
$IMPUC_t$	Urban consumer good imports
$IMPRC_{i,t}$	Rural consumer good imports
$IMPUI_t$	Urban investment good imports
$IMPRI_{i,t}$	Rural investment good imports
$MIGRATE_{i,t}$	Rural to urban migration from region i
$NETIN_{i,t}$	Nutrient flow
$NUTSUM_{i,t}$	Indexed accumulation of nutrient flows
$PEXP_{p,t}$	Public service expenditures in sector p
$PUBINV_t$	Total public investment

$RECCOST_{p,t}$	Recurrent public service expenditures in sector
$ROADCOST_t$	Total road investment and maintenance cost
$ROADINV_t$	Total road investment cost across regions
$ROADWAGE_{i,t}$	Road building wage across regions
$RWGOVR_{i,t}$	Real wage in rural government net of food
$RWGOVU_t$	Real wage in urban government net of food
$RWMAN_t$	Real wage manufacturing sector net of food
$SAVGNPWF_t$	Total saving as a share of GNP with food
$SAVR_{i,t}$	Rural gross saving in region
$SAVTOT_t$	Total gross saving
$SAVU_t$	Urban gross saving
TAX_t	Total tax revenues
TB_t	Trade balance
$TOTEXP_t$	Total expenditures across sectors in each period
$TOTIMPG_t$	Total government public service sector imports
$YDIS_t$	Disposable income (total population)
$YGOVR_{i,t}$	Real rural public sector wage net of food

Supplementary Online Appendix S3: Model Parameters

This paper focuses on the presentation of a general economic model rather than precise point estimates. Future research could usefully identify more refined subnational parameter values for Uganda. The parameters used for this model are presented below, and are set to a general baseline of the year 2002. Factor shares in production functions are assumed at standard values (e.g., see Eberhardt 2006). The cost of capital, depreciation rates, the FDI multiplier, share of services in consumption, and public sector service wage premiums are assumed. Capital stocks and productivity terms were estimated using the Uganda social accounting matrix for 2002 (Alarcon et al. 2006), and then scaled. Public sector import contents were set at one for simplicity.

The labor force is set to match the approximate reality of 19.6 million workers as of 2002, and the population of origin from each region matches the approximate real distribution of populations. The distribution of roads and agricultural capital stocks across the four regions provides another approximate match of the reality in Uganda. The northern region is poorest, and therefore starts the first period with slightly higher transport losses, lower cash crop capital stock, and lower cash crop productivity.

Sets:

i	Rural regions [east, west, central, north]
p	Public service sectors [health, education, general infrastructure, public administration]
reg	Geographic divisions [east, west, central, north, Kampala]
t	Time periods [T = 10]

Scalars:

Label	Description	Value
alphacc	labor share in cash crop production	0.4
alphaf	labor share in food production	0.5
alpham	labor share in manufacturing production	0.7
alphas	labor share in services	0.7
betacc	capital share in cash crop production	0.3
betaf	capital share in food production	0.2
cmin	minimum nonfood consumption	0.1
dep	depreciation	0.03
depcc	cash crop depreciation	0.03
fdimult	multiplier for FDI	0.1
gammas	share of services in the disposable income basket	0.5
km0	initial capital in manufacturing	10
ksu0	initial capital in urban services	10
kcscale	scaling parameter on CC capital stock	0.2
khurdle	hurdle parameter on household CC constraint	0.03
kmscale	scaling parameter on M capital stock	0.2
kroadscale	scaling parameter on road stock	0.25
ksscale	scaling parameter on SR capital stock	0.2
maintlabor	road maintenance labor-capital ratio	0.005
migtheta	rural-urban income migration parameter	0.015
nlossrate	nutrient loss rate in soil	0.03
phi	food requirement per person	0.55
pwageprem	public sector service wage premium	1.2
rho	logistic function parameter	1.5
rworld	world cost of capital	0.1
rsavcc	share of rural saving to cash crop	0.6
savurb	urban saving rate out of disposable income	0.3
savrur	rural saving rate out of disposable income	0.25
taxr	tax rate on disposable income	0.15
usavm	share of urban saving to manufacturing	0.5

Parameters:

(A) Scenario-based

$\boldsymbol{greenoda_t}$	ODA for Green Revolution Package
Scenario 1, 2:	$greenoda_t = 0$
Scenario 3:	{t = 1, 0; t = 2, 0; t = 3, 0.3; t = 4, 0.25; t = 5, 0.2; t = 6, 0.15; t = 7, 0.1; t = 8, 0; t = 9, 0; t = 10, 0}

(B) General

$\boldsymbol{impcontc_p}$	Import content share in commodities for public sector *p*
	$impcontc_p = 1$
$\boldsymbol{impcontk_p}$	Import content share in capital for public sector *p*
	$impcontk_p = 1$
$\boldsymbol{ltot_t}$	Total labor
	$ltot_t = 19.6$
$pimpr_t$	Price of imported goods in rural area
	$pimpr_t = 1$
$pimpu_t$	Price of imported goods in urban area
	$pimpu_t = 1$

(C) Regional

$\boldsymbol{commod_{p,reg,t}}$	Commodity expenditures in sector p in period t<br>$commod_{p,reg,t} = 0.01$
$\boldsymbol{invroad_{i,t}}$	Investment in rural road i in period t<br>$invroad_{i,t} = 0.2$
$\boldsymbol{invr_{p,i,t}}$	Rural public investment in sector p in period t<br>$invr_{p,i,t} = 0.04$
$\boldsymbol{invu_{p,t}}$	Urban public investment in sector p in period t<br>$invu_{p,t} = 0.04$
$\boldsymbol{kcc0_i}$	Initial capital in cash crops<br>{east 5, west 5, north 4, central 6}
$\boldsymbol{kf_i}$	Capital in food production<br>{east 5, west 6, north 5, central 4}
$\boldsymbol{ksr0_i}$	Initial capital in rural services<br>{east 5, west 5, north 5, central 5}
$\boldsymbol{klroad_i}$	K-L ratio for road building<br>{east 0.02, west 0.02, north 0.02, central 0.02}
$\boldsymbol{kpu0_p}$	Initial urban government capital stock in sector p<br>{health 1, educ 1, infr 1, padmin 1}
$\boldsymbol{kpr0_{i,p}}$	Initial rural capital stock in region i and sector p<br>$kpr0_{i,p} = 0.1$
$\boldsymbol{land_i}$	Land area in each rural region<br>{east 1.45, west 1.45, north 1.45, central 1.45}
$\boldsymbol{lpr_{p,i,t}}$	Rural labor in public sector p in period t<br>$lpr_{p,i,t} = 0.02$
$\boldsymbol{lpu_{p,t}}$	Urban labor in public sector p in period t<br>$lpu_{p,t} = 0.005$
$\boldsymbol{lr0_i}$	Initial rural labor<br>{east 4.5, west 4.5, north 3.8, central 4.5}
$\boldsymbol{nutrient0_i}$	Initial soil nutrient value<br>{east 1, west 1, north 1, central 1}
$\boldsymbol{pop_i}$	Population of origin from each region<br>{east 5.21, west 5.17, north 4.11, central 5.11}
$\boldsymbol{road0_i}$	Initial road value<br>{east 4, west 4, north 4, central 4}
$\boldsymbol{tlossfirst_i}$	Transport loss parameter<br>{east 0.5, west 0.55, north 0.6, central 0.45}
$\boldsymbol{thetacc_i}$	Cash crop sector productivity term<br>{east 1, west 1.1, north 0.8, central 1}
$\boldsymbol{thetaf_i}$	Food sector productivity term<br>{east 1, west 1, north 1, central 1}
$\boldsymbol{thetam_t}$	Manufacturing productivity (urban only)<br>$thetam_t = 1.25$
$\boldsymbol{thetasr_i}$	Rural service productivity<br>{east 1, west 1, north 1, central 1}
$\boldsymbol{thetasu_t}$	Urban service productivity:<br>$thetasu_t = 1.2$
$\boldsymbol{uppersoil_i}$	Upper soil productivity threshold<br>{east 1.552, west 1.552, north 1.552, central 1.552}

Supplementary Online Appendix S4: Overview of Uganda as Illustrative African Economy

Uganda faces many core challenges common across low-income African subsistence economies. Some key characteristics are described here. These draw from a range of sources, mainly published during the course of the early 2000s, and thus present a thematic overview rather than a precise snapshot at a single point in time. The data also predate Uganda's recent commencement of oil production.

As of 2012, approximately 35 percent of Uganda's population still lived below the international extreme poverty line of $1.90 per day in 2011 purchasing power parity terms (World Bank 2017). The vast majority of the country's poverty is concentrated in rural areas, where most Ugandans are engaged in crop agriculture. Infrastructure is limited. Only approximately 10 percent of households had electricity as of the early 2000s (Okidi et al. 2005). For decades, gross domestic saving rates were extremely low, well below 10 percent of GDP, although they averaged a slightly higher 13.1 percent from 2006 to 2015 (World Bank 2017). A 1997 Bank of Uganda survey found that fewer than a quarter of rural Ugandans had ever saved and that 85 percent of the other three-quarters cited low income as the primary factor for not doing so (Musinguzi and Smith 2000).

Staple Food Agriculture

Uganda's staple agriculture sector has experienced general long-term stagnation. Figure S4.1 presents trend data for cereal production per capita from 1961 to 2014. From a peak of nearly 180 kilograms per person in 1969, output has been stagnant, at less than 100 kilograms per person, since the early 1980s. Figure S4.2 indicates similar trends for a broader index of food production per capita.

Siriri, Bekunda, and Jama (2005) find that yields are typically one-quarter to one-tenth of current potential. This is significantly driven by the low usage of modern farm inputs. Fewer than a third of agricultural households use improved seeds, and only 8 percent use inorganic fertilizer (Okoboi and Barungi 2012). Yet despite the stagnation, Uganda has not become a marked food importer. Historically the coun-

Figure S4.1. Uganda Cereal Production per capita, 1961–2014

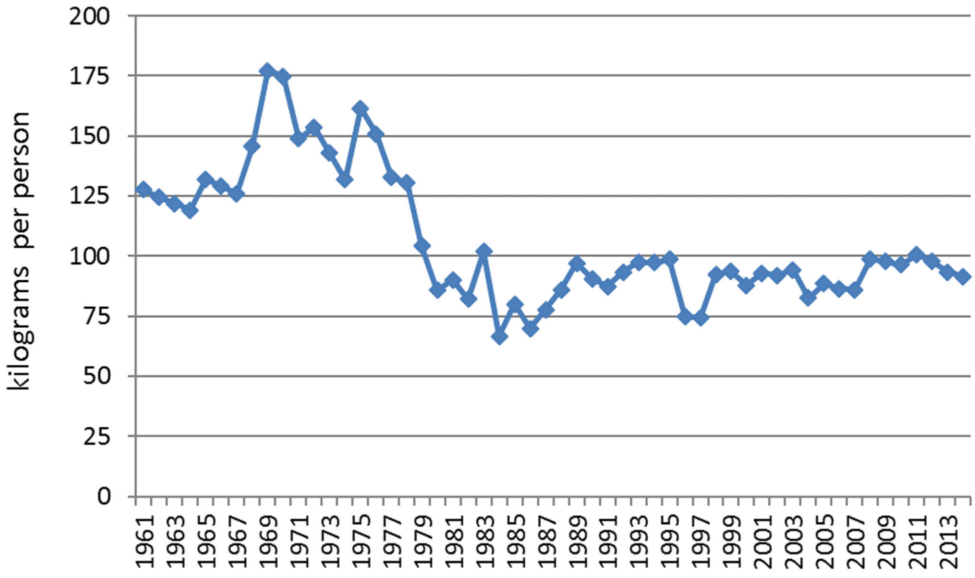

Source: World Bank (2017).

Figure S4.2. Uganda Food Production per capita, 1961–2014

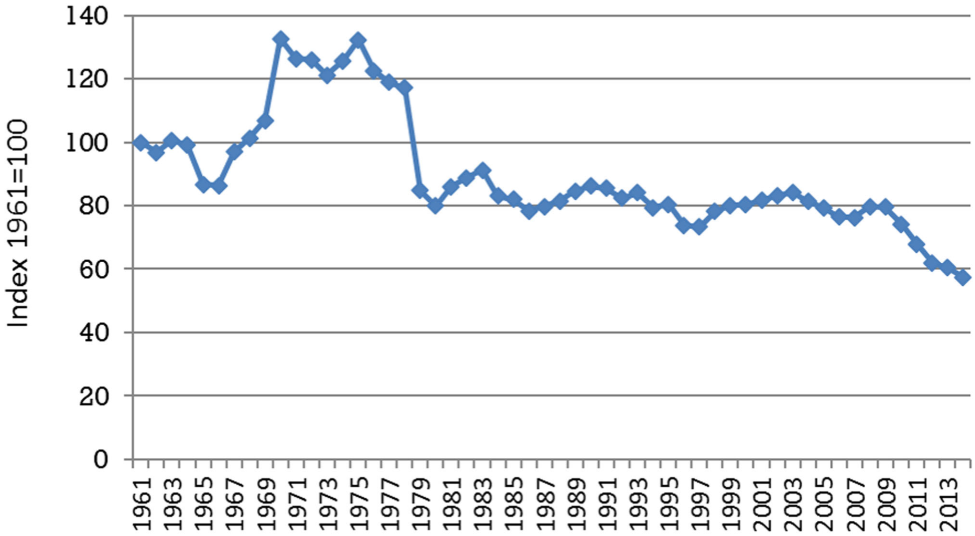

Source: World Bank (2017).

try has engaged in almost no staple crop trade; for many years nearly all its imported food was wheat and maize aid for conflict-affected areas in the north.

Soil Nutrients

Like much of Africa, Uganda faces a major soil nutrient challenge. For many years, agricultural output was maintained through land clearing, but population pressures (see Figure S4.3) and a lack of fallowing mean that farmers are now mining nutrients at faster rates and decreasing long-term yields in the process. Data compiled by Ssali (2002) and Ruecker (2005) indicate that a large portion of Uganda's soil is now below the so-called critical 3 percent value for soil organic matter. Henao and Baanante (2006) estimate loss rates for nitrogen, phosphorous, and potassium to be among the highest in Africa, at more than 60 kilograms per hectare per year. The consequences are significant. Nkonya, Kaizzi, and Pender (2005) estimate a cost of approximately $153 per household per year to replenish mined soil nutrients at market prices, equivalent to nearly a fifth of GDP at the time of calculation.

Fallow periods have fallen from 10 to 15 years a century ago down to two and even zero years today (Nandwa and Bekunda 1998). In large parts of the country, fewer than 10 percent of farms have been estimated even to use fallows (Pender et al. 2001). Fertilizer is necessary, even if not sufficient, to stop and reverse the patterns of nutrient decline and address the soil nutrient challenge. However, cost is a barrier because staple crop farmers often face poor relative returns on fertilizer, often with a "value to cost ratio" of 1 or less (e.g., Wortmann and Kaizzi 1998; de Jager, Onduru, and Walaga 2003; Kaizzi 2002; Matsumoto and Yamano 2009).

Uganda's internal geographic heterogeneity underscores the disparate range of farming systems across Africa. Ruecker et al. (2003) identified seven categories of agricultural potential across the country based on permutations of four climate variables. First, annual precipitation cycles affect the extent of water availability throughout the year. The northeastern section of the country has unimodal rainfall, while the southern and central areas, which are closer to the equator, have bimodal rainfall. Second, the length of growing period is measured as the period over which mean monthly rainfall exceeds half the mean

Figure S4.3. Uganda Arable Land per person, 1961–2014

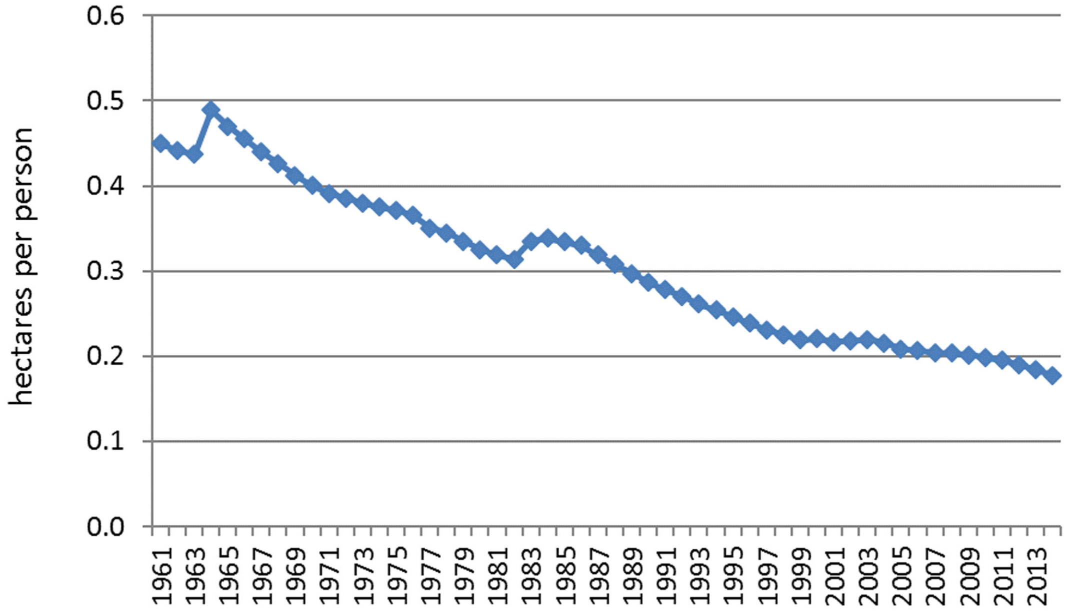

Source: World Bank (2017).

potential evapotranspiration. This ranges from less than five months in the northeastern districts to 10 or more months in the central region and in the southwestern highlands. Third, the actual level of annual precipitation varies tremendously throughout the country and changes at a steep gradient, particularly in the "crescent" around Lake Victoria. Fourth, extreme temperatures constrain agricultural productivity. The range of growing conditions results in significant variations in the concentration of staple crop by region.

Other Key Sectors

Cash crops, especially coffee, have historically been a major driver of Uganda's growth and poverty reduction. However, fewer than 10 percent of the country's farm households grow coffee, and the commodity's share of exports has declined significantly (Kappel, Lay, and Steiner 2005; Bussolo et al. 2006). As of the mid-2000s, cotton, tea, and tobacco had increased in volume and total value exported, while flower exports had also been introduced. Fisheries have overtaken coffee in overall export value, but only a small share of the population is engaged in that activity. In any case, all the export shares need to be considered in the context of Uganda's low overall export/GDP ratio, which ranged between 15 and 24 percent from 2006 to 2015 (World Bank 2017).

Manufacturing remains a small element of the economy, historically accounting for less than 10 percent of GDP, as indicated in table S4.1. Table S4.2 shows that the sector still employed less than 4 percent of the labor force as of 2002. The service sectors—including wholesale and retail trade, transport, communications, and construction—employed approximately 9 percent of the labor force, split fairly evenly between urban and rural areas, accounting for approximately one-third of GDP. Government accounted for the largest share of GDP, at approximately 20 percent, and 7 percent of the labor force. Most of the public labor force is situated in rural areas where the population lives, but approximately one-third is based in urban areas, especially the public administration hub of Kampala.

Table S4.1. Uganda GDP Sector Shares, 1990/91–2004/05

	1990/91	1994/95	1999/00	2004/05
MONETARY SECTORS				
Agriculture	22.7%	25.2%	20.3%	18.7%
- Cash crops	2.8%	6.3%	3.6%	2.8%
- Food crops	12.3%	12.2%	10.7%	10.2%
- Livestock	4.1%	3.4%	3.4%	2.7%
- Forestry	1.1%	0.9%	0.7%	0.7%
- Fishing	2.4%	2.3%	2.0%	2.3%
Mining & quarrying	0.3%	0.3%	0.8%	0.9%
Manufacturing	5.7%	7.2%	9.4%	9.0%
Electricity/water	0.9%	1.3%	1.4%	1.3%
Construction	4.8%	5.5%	7.6%	9.3%
Wholesale & Retail Trade	12.3%	12.0%	11.2%	10.9%
Hotels & Restaurants	1.3%	1.8%	2.4%	3.3%
Transport/communication	4.0%	3.9%	5.3%	7.7%
- Road	2.9%	3.0%	3.7%	3.5%
- Rail	0.3%	0.2%	0.2%	0.1%
- Air & Support. Services	0.4%	0.4%	0.5%	0.5%
- Communications	0.4%	0.4%	0.9%	3.6%
Community services	15.4%	17.0%	20.4%	20.0%
- General government	3.2%	4.5%	4.3%	4.2%
- Education	3.9%	4.0%	6.1%	6.4%
- Health	1.5%	1.3%	2.2%	2.3%
- Rents	3.2%	3.5%	4.3%	3.8%
- Miscellaneous	3.6%	3.8%	3.6%	3.2%
**TOTAL MONETARY**	**67.4%**	**74.1%**	**78.7%**	**81.0%**
NON-MONETARY SECTORS				
Agriculture	28.4%	22.3%	16.9%	14.8%
- Food crops	25.7%	19.9%	13.7%	11.9%
- Livestock	1.3%	1.2%	1.9%	1.7%
- Forestry	1.1%	0.9%	1.2%	1.0%
- Fishing	0.3%	0.3%	0.3%	0.3%
Construction	0.8%	0.7%	0.6%	0.5%
Owner-occupied Dwelling	3.3%	2.9%	3.7%	3.7%
**TOTAL NON-MONETARY**	**32.6%**	**25.9%**	**21.3%**	**19.0%**

Source: Authors' calculations based on data from Uganda Ministry of Finance, Planning, and Economic Development (2005).

Transport Costs and Infrastructure

A key attribute of Uganda's economy is its limited infrastructure and high transport costs, which are among the highest in the world (Buys 2006). In multiple sectors, including food, these costs provide both implicit protection for domestic producers and implicit tax on exports (Milner, Morrissey, and Rudaheranwa 2000; Rudaheranwa 2006). Poor roads are responsible for much of the high costs. The 2005 National Transport Plan reported only 5 percent of the country's roads as paved, and only approximately 40 percent of those in good condition (Uganda Ministry of Works, Housing, and Communications and TAHAL Engineers 2005).

Table S4.2. Sectoral Decomposition of Ugandan Labor Force and Value Added, 2002

Sector	Rural labor	% of national labor force	Urban labor	% of national labor force	Total labor	% of national labor force	% of Net Value Added
Staples and Cash Crops	4,796,824	69.0%	115,768	1.7%	4,912,592	70.7%	18.3%
Animal farming	260,581	3.7%	22,298	0.3%	282,879	4.1%	2.1%
Mining and Quarrying	14,616	0.2%	5,124	0.1%	19,740	0.3%	0.3%
Tradable Manufacturing	208,296	3.0%	64,737	0.9%	273,033	3.9%	10.6%
Utilities	5,508	0.1%	8,861	0.1%	14,369	0.2%	3.9%
Non-tradable local capital goods	58,774	0.8%	49,934	0.7%	108,708	1.6%	11.6%
Non-tradable services	190,815	2.7%	222,521	3.2%	413,336	5.9%	28.4%
Tradable services	105,806	1.5%	117,219	1.7%	223,025	3.2%	8.1%
Public service	317,228	4.6%	188,741	2.7%	505,969	7.3%	16.7%
Other	99,193	1.4%	97,193	1.4%	196,386	2.8%	
Totals	6,057,641	87.2%	892,396	12.8%	6,950,037	100.0%	100.0%

Source: Authors' calculations based on data from Uganda Bureau of Statistics (2004) and Alarcon et al. (2006).

References

Alarcon, J., S. Baryahirwa, S. Bahemuka, J. Van Heemst, I. Kasirye, N. Rudaheranwa, and P. de Valk. 2006. "Social Accounting Matrix Uganda 2002—Project Report: Output, Procedures and Methodology." Economic and Policy Research Centre (EPRC), Institute for Social Studies (ISS), Uganda Bureau of Statistics (UBOS), The Hague, Netherlands.

Bussolo, M., O. Godard, J. Lay, and R. Thiele. 2006. "The Impact of Commodity Price Changes on Rural Households: The Case of Coffee in Uganda." Policy Research Working Paper No. 4088, World Bank, Washington, DC.

Buys, P. 2006. "Road Network Upgrading and Overland Trade Expansion in Sub-Saharan Africa." Policy Research Working Paper No. 4097, World Bank, Washington, DC.

de Jager, A., D. Onduru, and C. Walaga. 2003. "Facilitated Learning in Soil Fertility Management: Assessing Potentials of Low-External-Input Technologies in East African Farming Systems." *Agricultural Systems* 79 (2): 205–23.

Eberhardt, M. 2006. "Technical Progress and Structural Change as Sources of Productivity Growth: Some Macroeconomic Evidence." MPhil thesis. Department of Economics, University of Oxford.

Henao, J., and C. Baanante. 2006. *Agricultural Production and Soil Nutrient Mining in Africa: Implications for Resource Conservation and Policy Development*. Muscle Shoals, AL: International Center for Soil Fertility and Agricultural Development.

Kaizzi, C. K. 2002. "The Potential Benefit of Green Manures and Inorganic Fertilizers in Cereal Production on Contrasting Soils in Eastern Uganda." PhD thesis, University of Bonn and German Center for Development Research (ZEF).

Kappel, R., J. Lay, and S. Steiner. 2005. "Uganda: No More Pro-Poor Growth?" *Development Policy Review* 23 (1): 27–53.

Matsumoto, T., and T. Yamano. 2009. "Soil Fertility, Fertilizer, and the Maize Green Revolution in East Africa." Policy Research Working Paper No. 5158, World Bank, Washington, DC.

Milner, C., O. Morrissey, and N. Rudaheranwa. 2000. "Policy and Non-Policy Barriers to Trade and Implicit Taxation of Exports in Uganda." *Journal of Development Studies* 37 (2): 67–90.

Musinguzi, P., and P. Smith. 2000. "Saving and Borrowing in Rural Uganda." Department of Economics, Discussion Papers in Economics and Econometrics, University of Southampton, Southampton, UK.

Nandwa, S. M., and M. A. Bekunda. 1998. "Research on Nutrient Flows and Balances in East and Southern Africa: State-of-the-Art." *Agriculture, Ecosystems and Environment* 71 (1-3): 5–18.

Nkonya, E., C. Kaizzi, and J. Pender. 2005. "Determinants of Nutrient Balances in a Maize Farming System in Eastern Uganda." *Agricultural Systems* 85 (2): 155–82.

Okidi, J. A., S. Ssewanyana, L. Bategeka, and F. Muhumuza. 2005. *Operationalizing Pro-Poor Growth*. Kampala: Economic Policy Research Centre.

Okoboi, G., and M. Barungi. 2012. “Constraints to Fertiliser Use in Uganda: Insights from Uganda Census of Agriculture 2008/9.” Research Series No. 88, Economic Policy Research Centre, Kampala.

Pender, J., P. Jagger, E. Nkonya, and D. Sserunkuuma. 2001. “Development Pathways and Land Management in Uganda: Causes and Implications.” EPTD Discussion Paper No. 85, International Food Policy Research Institute, Washington, DC.

Rudaheranwa, N. 2006. “Trade Policy and Transport Costs in Uganda.” CREDIT Research Paper No. 06/09, University of Nottingham, Nottingham, UK.

Ruecker, G. R. 2005. “Spatial Variability of Soils on National and Hillslope Scale in Uganda.” Ecology and Development Series No. 24, University of Bonn ZEF, Bonn.

Ruecker, G. R., S. Park, H. Ssali, and J. L. Pender. 2003. “Strategic Targeting of Development Policies to a Complex Region: A GIS-Based Stratification Applied to Uganda.” Discussion Papers on Development Policy No. 69, University of Bonn ZEF, Bonn.

Siriri, D., M. Bekunda, and B. Jama. 2005. “Limiting Soil Nutrients in Selected Farming Systems of Uganda.” Unpublished paper. Kampala, Uganda.

Ssali, H. 2002. “Presentation to Uganda Parliamentary Forum: November 2002.” Report presented to Uganda Parliamentary Forum, Kampala, Uganda, November.

Uganda Bureau of Statistics. 2004. Uganda Census 2002. Entebbe: Uganda Bureau of Statistics.

Uganda Ministry of Finance, Planning, and Economic Development. 2005. Macroeconomic Data. Entebbe: Uganda Bureau of Statistics.

Uganda Ministry of Works, Housing, and Communications and TAHAL Engineers. 2005. Uganda National Transport Plan. Entebbe: Uganda Bureau of Statistics.

World Bank. 2017. World Development Indicators. Online database (accessed November 15, 2017), http://databank.worldbank.org/data/reports.aspx?source=world-development-indicators#.

Wortmann, C. S., and C. K. Kaizzi. 1998. “Nutrient Balances and Expected Effects of Alternative Practices in Farming Systems of Uganda.” *Agriculture, Ecosystems and Environment* 71 (1-3): 115–29.
